# Supplementary material for: Direct dehydrocoupling facilitates efficient thiophene anchoring on silicon surfaces
Source: Nat Commun. 2025 Jul 19;16:6664. doi: 10.1038/s41467-025-62002-7 (PMC12276279; doi:10.1038/s41467-025-62002-7)
Supplement: Supplementary file 1 — Supplementary Information [file 41467_2025_62002_MOESM1_ESM.pdf]

Supplementary Materials for

Direct Dehydrocoupling Facilitates Efficient Thiophene Anchoring on Silicon Surfaces

Jingpeng Li,[1] Meiyu Zhang,[1] Wenxuan Li,[1] Zhongshu Li[1], Tingshun Zhu[1], and Zhenyu Yang[1]*

1. MOE Laboratory of Bioinorganic and Synthetic Chemistry, Lehn Institute of Functional Materials, School of Chemistry, IGCME, Sun Yat-sen University, Guangzhou, Guangdong, 510275, China

Corresponding E-mail: yangzhy63@mail.sysu.edu.cn

KEYWORDS: silicon, dehydrocoupling, radical reaction, surface chemistry, photophysics

Supplementary Note 1 | ^{t}BuOK-catalyzed thiophene-anchoring reaction on SiNC surfaces.

~15 mg of H-SiNC was diluted to 5 mL with THF and transferred into a 25-mL reaction tube. 0.268 g (2 mmol) of 1-benzothiophene (BT) and 44 mg (0.4 mmol) of ^{t}BuOK were weighed within a nitrogen-filled glovebox and transferred to the reaction tube immediately. The reaction tube was then refilled with nitrogen, and the temperature was set to 45°C to initiate the reaction. After 24 h, the reaction tube was naturally cooled to room temperature. The subsequent purification steps are similar to BT-SiNC as outlined in Methods.

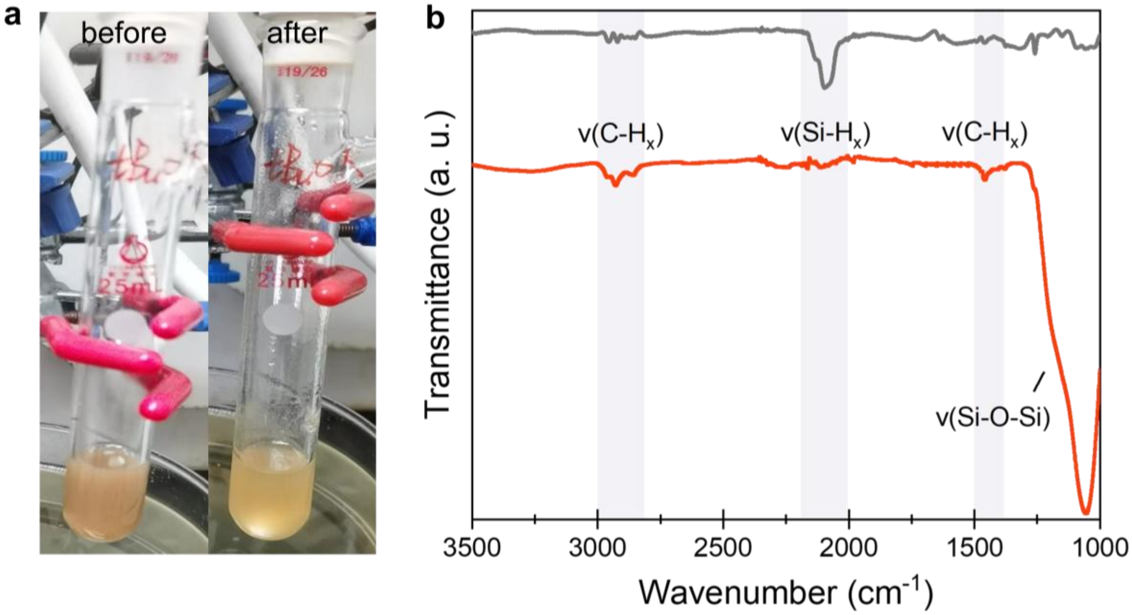

Supplementary Fig. 1 ^{t}BuOK-catalyzed thiophene-anchoring reaction on SiNC surface. (**a**) Image before and after the addition of ^{t}BuOK following 24 hours of reaction. (**b**) FT-IR spectra of SiNC before (grey) and after ^{t}BuOK-functionalization (red).

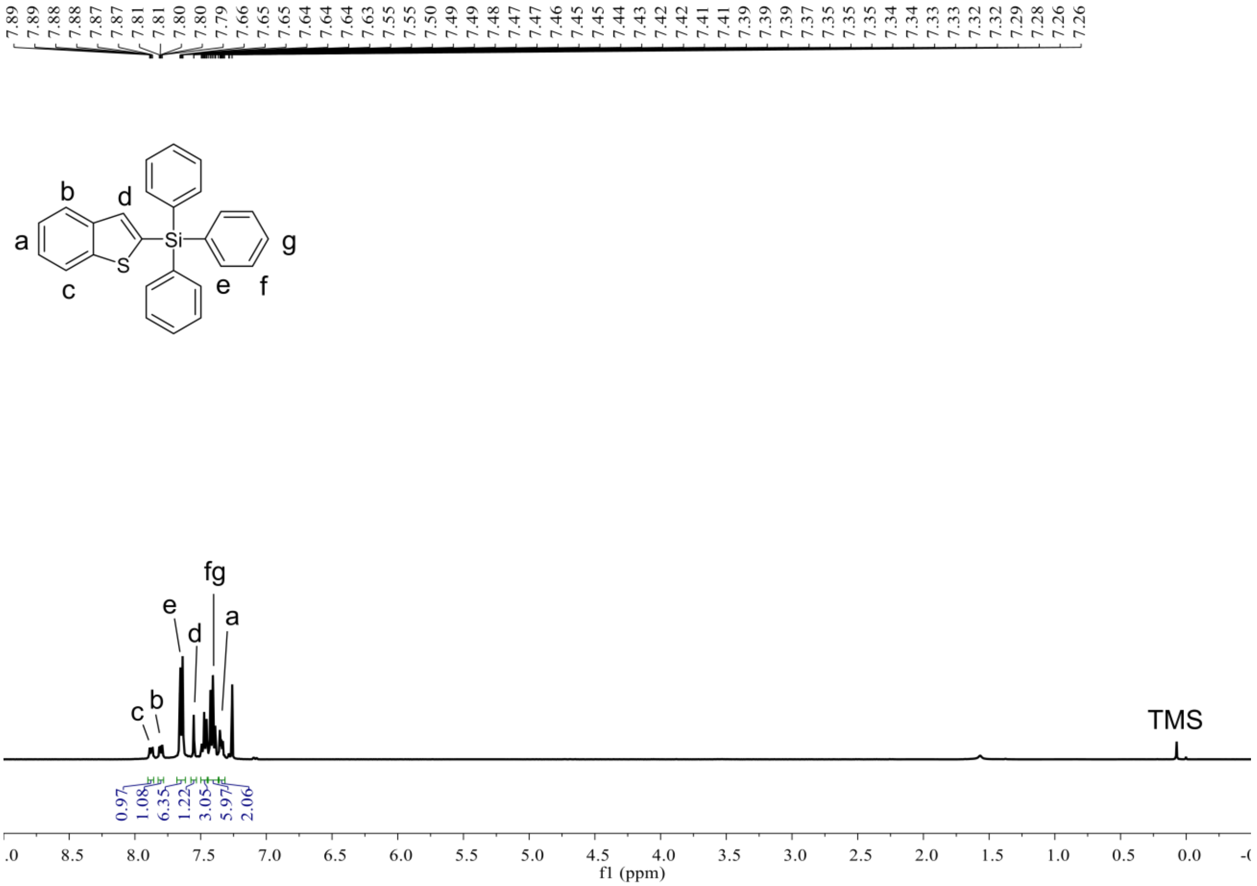

Supplementary Fig. 2 ^{1}H NMR spectrum of BT-TPS (400 MHz, chloroform-*d*). δ 7.26 (residual chloroform), 1.54 (trace amount of H_2O in solvent), 7.89 – 7.88 (t, *J* = 7.4 Hz, 1H, benzothiophene-ArH), 7.81 – 7.79 (t, 1H, *J* = 7.4 Hz, benzothiophene-ArH), 7.66 – 7.63 (m, 6H, triphenylsilane-Ar-H (Si-ArH) ×3), 7.55 (s, 1H, thiophene-H), 7.50 – 7.45 (m, 3H, Si-ArH ×3), 7.43 – 7.39 (m, 6H, Si-ArH ×3), 7.37 – 7.32 (m, 2H, benzothiophene-ArH).

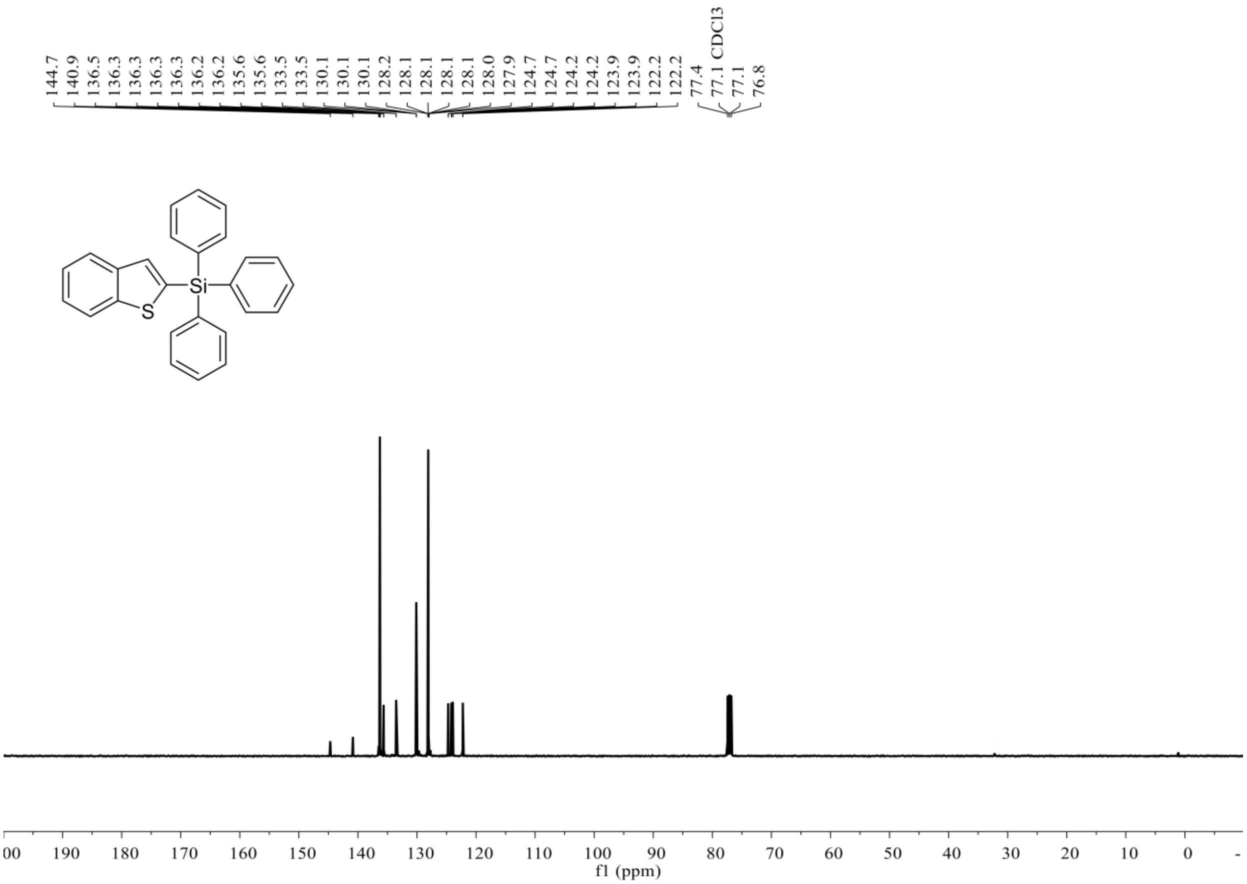

Supplementary Fig. 3 ^{13}C NMR spectrum of BT-TPS (100 MHz, chloroform-*d*). δ 77.16 (residual chloroform), 144.7 and 140.9 (benzothiophene-ArC), 136.5, 136.3 and 135.7 (Si-ArC), 133.5 (thiophene-C), 130.1, 128.1, 128.1, 124.7 and 124.2 (Si-ArC), 123.9 and 122.3 (benzothiophene-ArC).

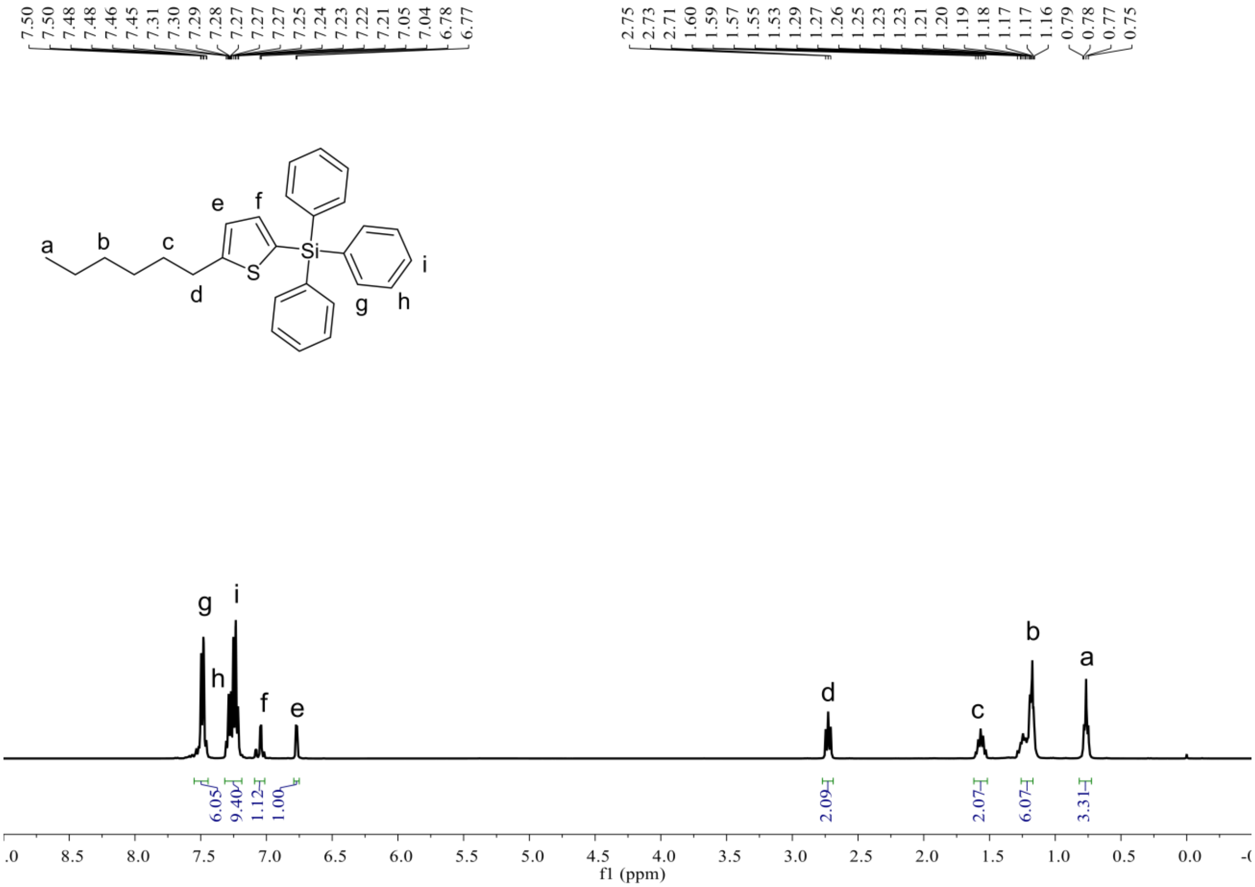

Supplementary Fig. 4 ^{1}H NMR spectrum of 1T-TPS (400 MHz, chloroform-*d*). δ 7.26 (residual chloroform), 1.50 (trace amount of H_2O in solvent), 7.48 – 7.44(m, 6H, Si-ArH ×3), 7.31 – 1.21 (m, J = 7.4 Hz, 9H, Si-ArH ×3), 7.05 (d, J = 3.4 Hz, 1H, thiophene-H), 6.78 (d, J = 3.3 Hz, 1H, thiophene-H), 2.73 (t, J = 7.7 Hz, 2H, thiophene-C$\underline{H}_2$CH$_2$(CH$_2$)$_3$CH$_3$), 1.60 – 1.53 (m, 2H, thiophene-CH$_2$C$\underline{H}_2$(CH$_2$)$_3$CH$_3$), 1.29 – 1.16 (m, 6H, thiophene-CH$_2$CH$_2$(C$\underline{H}_2$)$_3$CH$_3$), 0.79 – 0.75 (t, 3H, thiophene-CH$_2$CH$_2$(CH$_2$)$_3$C$\underline{H}_3$).

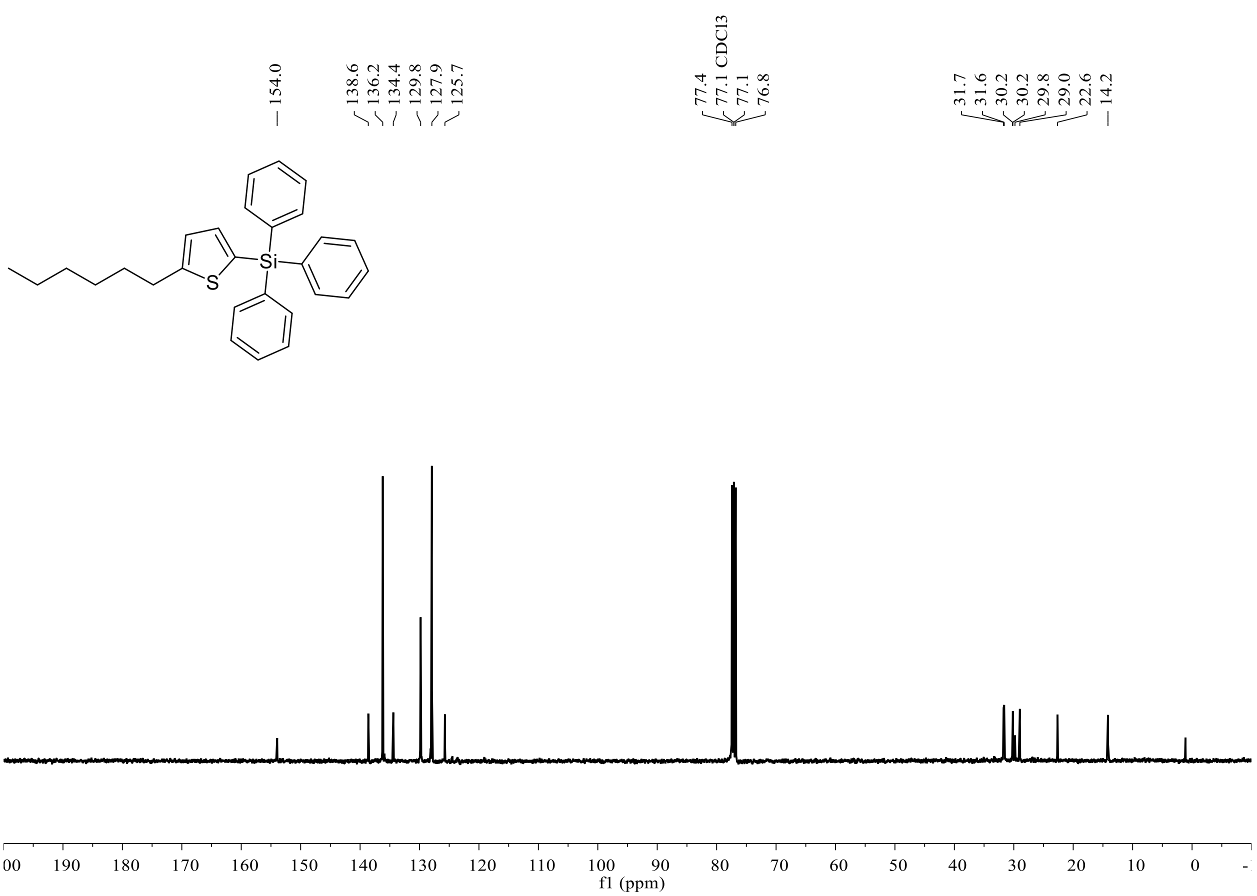

Supplementary Fig. 5 ^{13}C NMR spectrum of 1T-TPS (100 MHz, chloroform-*d*). δ 77.16 (residual chloroform), 153.9 (thiophene-C), 138.6, 136.2, 134.4 and 129.8 (Si-ArC), 127.9 and 125.7 (thiophene-C), 31.7 (thiophene-<u>C</u>$H_2CH_2(CH_2)_3CH_3$), 31.6 (thiophene-CH_2<u>C</u>$H_2(CH_2)_3$ CH_3), 30.2, 29.8 and 22.6 (thiophene-CH_2CH_2(<u>C</u>$H_2)_3CH_3$), 14.2 (thiophene-$CH_2CH_2(CH_2)_3$<u>C</u>H_3).

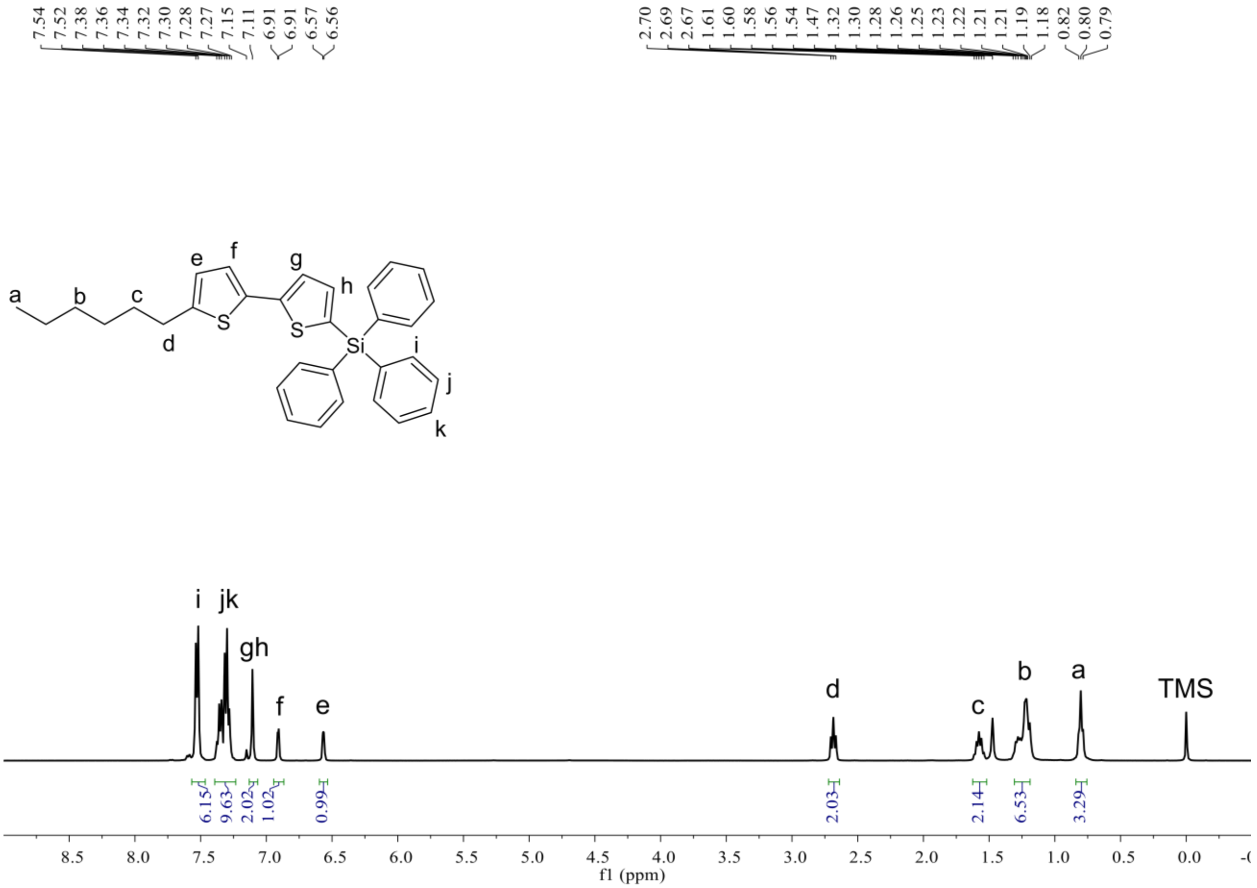

Supplementary Fig. 6 ^{1}H NMR spectrum of 2T-TPS (400 MHz, chloroform-*d*). δ 7.26 (residual chloroform), 1.50 (trace amount of H_2O in solvent), 7.53 (d, J = 7.2 Hz, 6H, Si-ArH ×3), 7.32 – 7.15 (m, 9H, Si-ArH ×3), 7.11 (s, 2H, thiophene-H), 6.91 (d, J = 3.5 Hz, 1H, thiophene-H), 6.57 (d, J = 3.5 Hz, 1H, thiophene-H), 2.69 (t, J = 7.6 Hz, 2H, thiophene-$C\underline{H}_2CH_2(CH_2)_3CH_3$), 1.62 – 1.52 (m, 2H, thiophene-$CH_2C\underline{H}_2(CH_2)_3CH_3$), 1.31 – 1.19 (m, 6H, thiophene-$CH_2CH_2(C\underline{H}_2)_3CH_3$), 0.84 – 0.76 (m, 3H, thiophene-$CH_2CH_2(CH_2)_3C\underline{H}_3$).

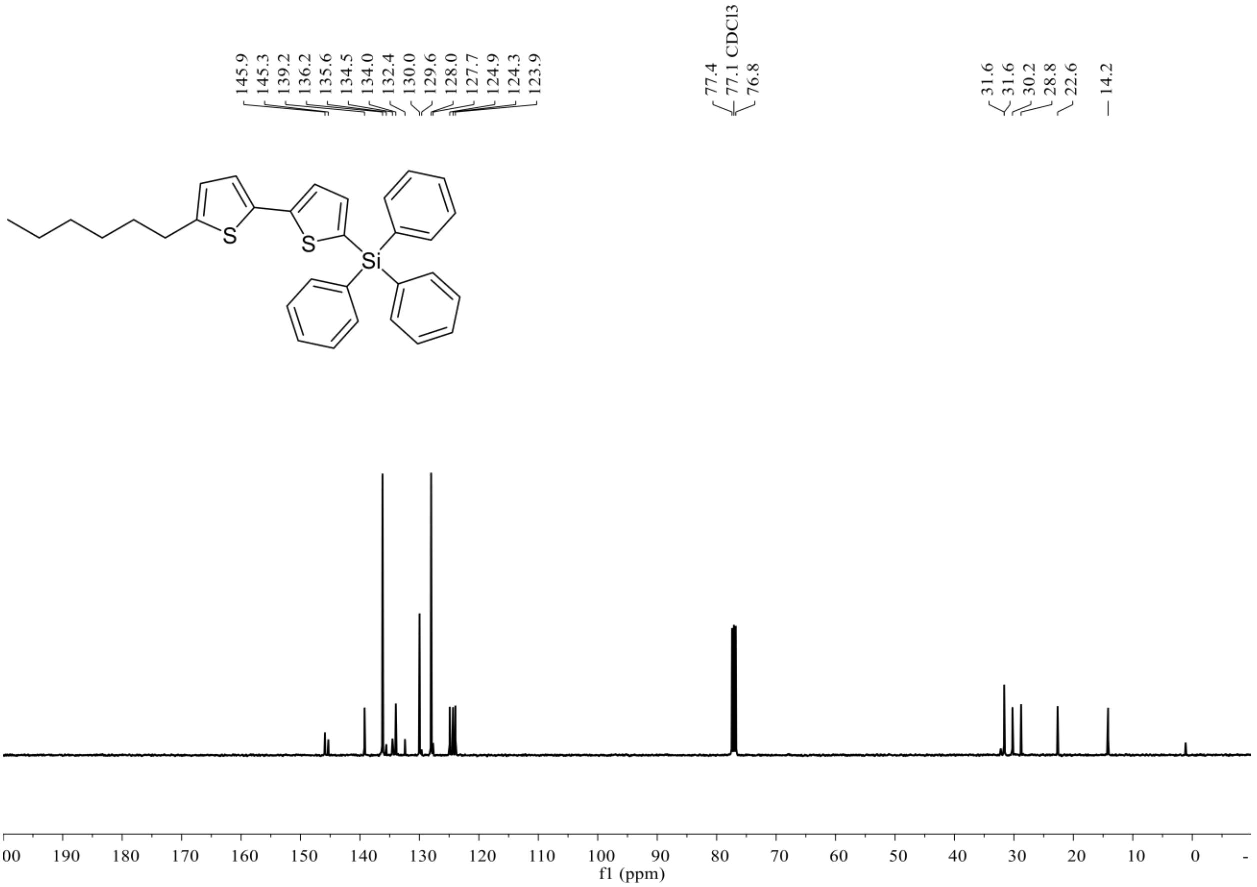

Supplementary Fig. 7 ^{13}C NMR spectrum of 2T-TPS (100 MHz, chloroform-*d*). δ 77.16 (residual chloroform), 145.9, 145.3 and 139.2 (thiophene-C), 136.2, 134.5, 133.9, 132.4, 129.9, and 128.0 (Si-ArC), 124.9, 124.3 and 123.9 (thiophene-C), 31.6 (thiophene-$\underline{C}H_2CH_2(CH_2)_3CH_3$), 31.6 (thiophene-$CH_2\underline{C}H_2(CH_2)_3CH_3$), 30.2, 28.8 and 22.7 (thiophene-$CH_2CH_2(\underline{C}H_2)_3CH_3$), 14.2 (thiophene-$CH_2CH_2(CH_2)_3\underline{C}H_3$).

Supplementary Table 1 | Summary of the X-ray single-crystal diffraction results of BT-TPS.

Identification	BT-TPS
Empirical formula	$C_{26}H_{20}SSi$
Formula weight	392.57
Temperature/K	222.99 (10)
Crystal system	monoclinic
Space group	$P2_1$
a/Å	6.94550(10)
b/Å	11.4351(2)
c/Å	13.1100(3)
α/°	90
β/°	96.621(2)
γ/°	90
Volume/Å^3	1034.28(3)
Z	2
ρ_{calc}g/cm^3	1.261
μ/mm^{-1}	1.990
F(000)	412.0
Crystal size/mm^3	0.15 × 0.08 × 0.06
Radiation	Cu Kα (λ = 1.54184)
2Θ range for data collection/°	6.788 to 133.178
Index ranges	$-8 \le h \le 8$, $-13 \leqslant k \leqslant 13$, $-14 \leqslant l \leqslant 15$
Reflections collected	8076
Independent reflections	3658 [R_{int} = 0.0487, R_{sigma} = 0.0579]
Data/restraints/parameters	3658/13/271
Goodness-of-fit on F^2	1.077
Final R indexes [I>=2σ (I)]	R_1 = 0.0535, wR_2 = 0.1352
Final R indexes [all data]	R_1 = 0.0583, wR_2 = 0.1395
Largest diff. peak/hole / e Å^{-3}	0.45/-0.26

Supplementary Table 2 | The average rate and related parameters of the reported direct dehydrogenation coupling reaction of silane and thiophenes were summarized.

Catalysis	Time (h)	Conversion (%)	Average Rate (10^{-3} mol ml^{-1} h^{-1})	Ref.# in ESI
$Cu(OAc)_2$	24	71	0.035	1
$Ni(cod)_2$	36	66	0.056	2
DTBPY	24	87	0.025	3
Au-(NPs)	3	97	0.104	4
CsF	24	81	0.042	5
ICIB	72	73	0.030	6
$Ni(cod)_2$	24	65	0.017	7
ScMPF	24	42	0.004	8
$[Rh(nbd)Cl]_2$	7	67	0.046	9
^{t}BuOK	40	97	0.050	10
**N/A**	**12**	**94**	**0.083**	**This work**

Note: $Cu(OAc)_2$ = Cupric Acetate Monohydrate; $Ni(cod)_2$ = 1,2,5,6-Cyclooctanetetraylidene, nickel salt; DTBPY = [{Ir(OMe)(cod)}2]/4,4-di-tert-butyl-2,2-bipyridine (cod=cycloocta-1,5-diene); Au-(NPs) = Au nanoparticles; CsF = Cesium fluoride; ICIB = $[Ir(acetone)(COD)(IPr)][BF_4]$ (COD =1,5-cyclooctadiene); ScMPF = Scandium alkyl complex(methyl-, and 2-*n*-propyl-furans); $[Rh(nbd)Cl]_2$ = Bicyclo[2.2.1]hepta-2,5-diene-rhodium chloride dimer; ^{t}BuOK = Potassium *t*-butoxide.

Supplementary Note 2 | Verification of radical-drive process of the dehydrogenative coupling between BT and TPS using radical trapping experiments.

1-benzothiophene (BT, 134 mg, 1.0 mmol) was combined with triphenylsilane (TPS, 284 mg, 1.1 mmol) and 2,2,6,6-tetramethylpiperidoxyl (TEMPO, 2.0 eq.) in a reaction tube and heated to 180°C for 12 h in the presence of air. Following this, a portion of the mixture was dissolved in methanol. The product signals assigned to the TEMPO incorporated silane structure (TEMPO-TPS) were found in the results of the high-resolution mass spectrum (HRMS), and the BT coupling TPS structure (BT-TPS) was not found, as shown in Supplementary Figure 8.

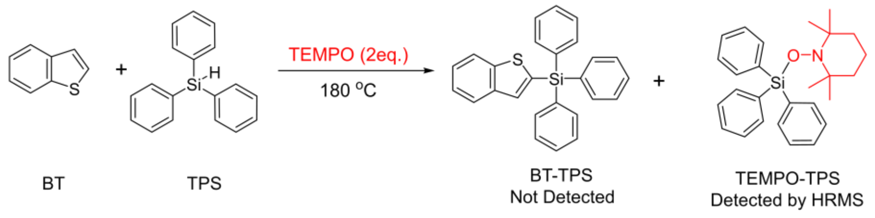

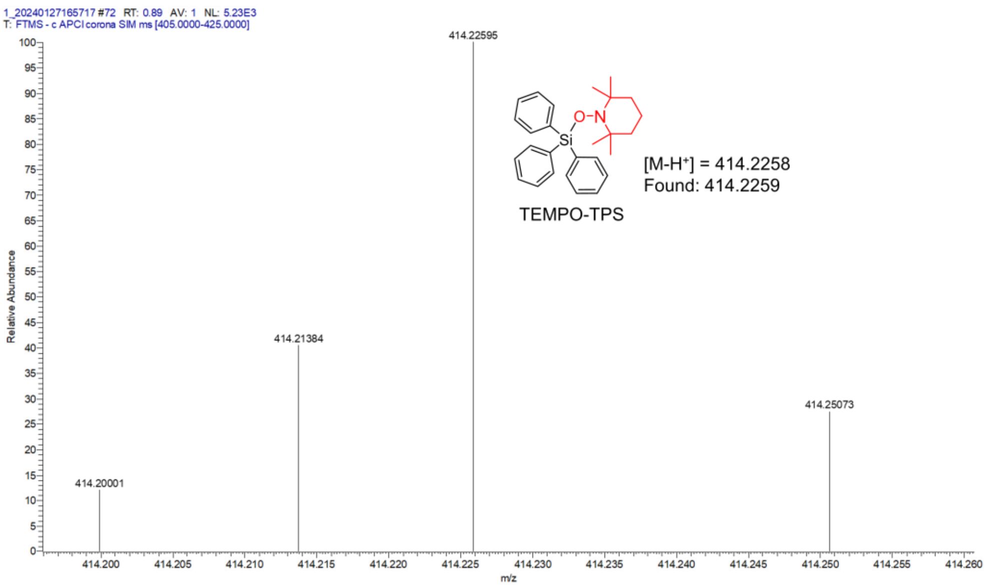

Supplementary Fig. 8 Reaction parameters and HRMS results of the interaction between BT and TPS with the presence of TEMPO.

Supplementary Note 3 | Detection of H_2 formed from the dehydrogenative coupling reaction between BT and TPS.

In a nitrogen-filled glovebox, 147 mg of 1-benzothiophene (BT, 1.1 mmol) and 259 mg of triphenylsilane (TPS, 1.0 mmol) were encapsulated in a 20 ml vial the bottle mouth was closed with a rubber stopper, and the periphery was wrapped with wire and tightened. The sample vials were removed from the glove box and heated at 180 °C for 12 h. The gas was withdrawn from the vials after cooling to room temperature and GC measured the gas composition.

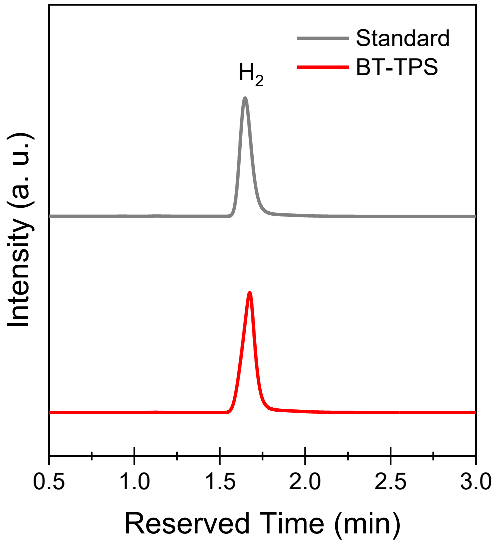

Supplementary Fig. 9 GC signal of H_2 from the mixture BT and TPS at 180 ^oC and the comparison with the standard H_2 signal.

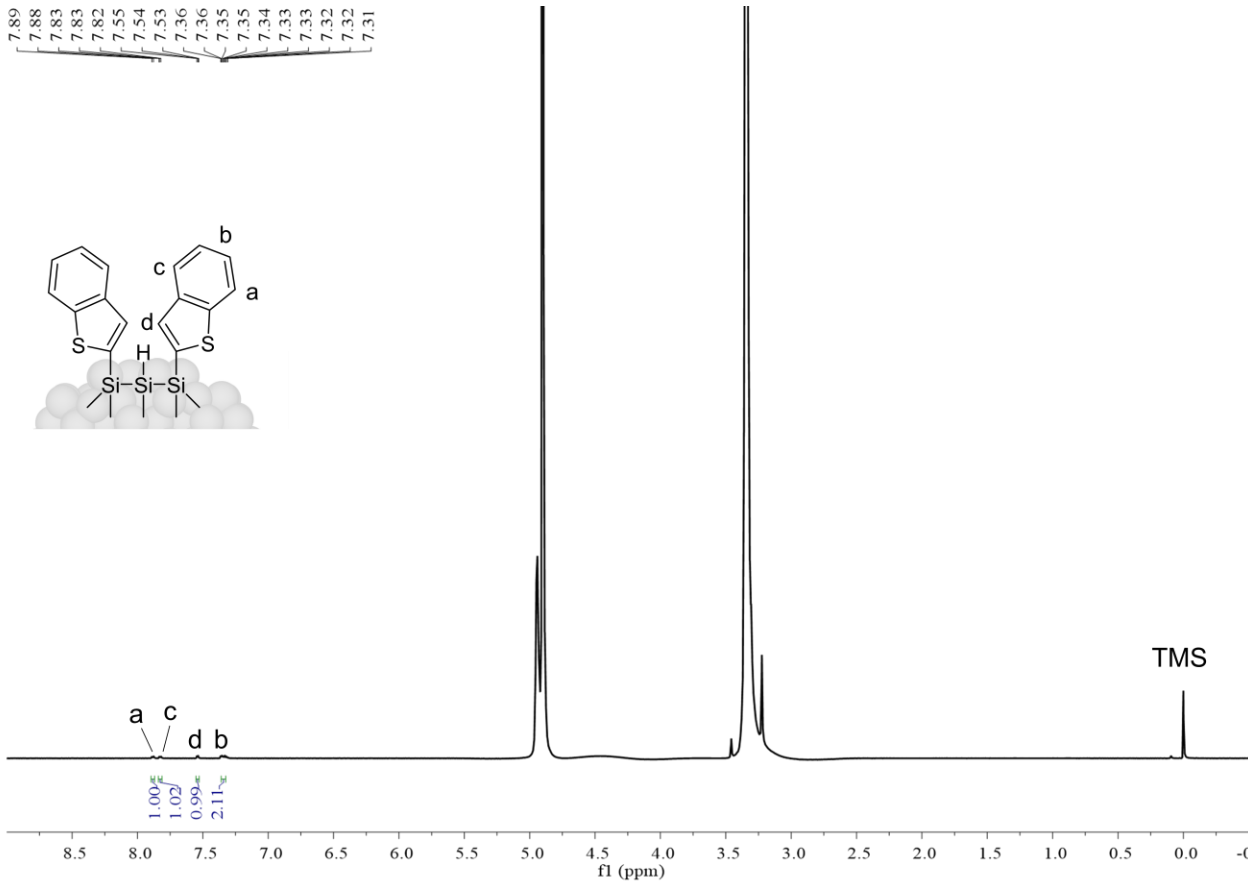

Supplementary Fig. 10 ^{1}H NMR spectrum of BT-SiNC (600 MHz, methanol-*d*$_4$). δ 3.31 (residual chloroform), 4.87 (trace amount of H_2O in solvent), 7.89 – 7.88 (t, J = 7.5 Hz, 1H, benzothiophene-ArH), 7.83 – 7.82 (t, J = 7.7 Hz, 1H, benzothiophene-ArH), 7.53 (s, 1H, thiophene-H), 7.36 – 7.31 (m, 2H, benzothiophene-ArH).

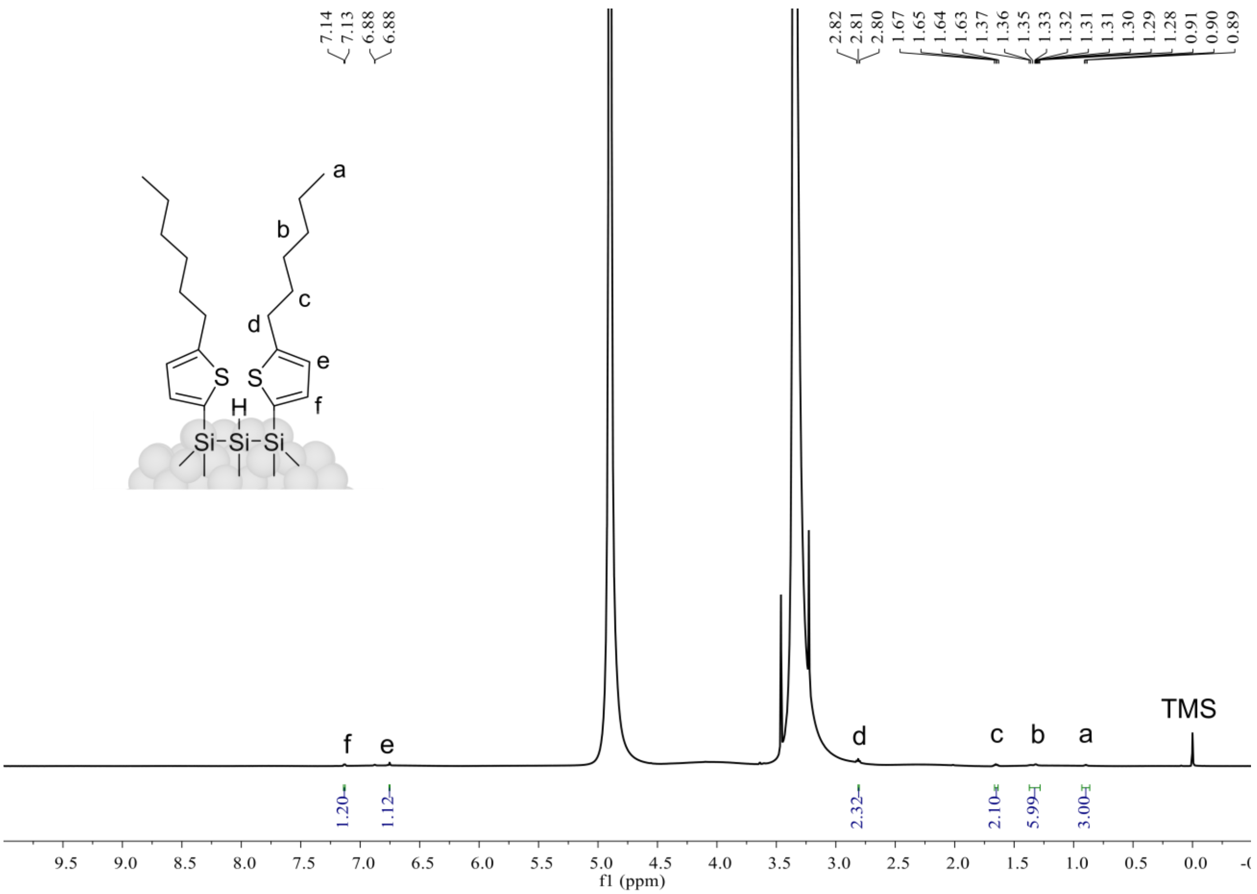

Supplementary Fig. 11 ^{1}H NMR spectrum of 1T-SiNC (600 MHz, methanol-*d*$_4$). δ 3.31 (residual chloroform), 4.87 (trace amount of H_2O in solvent), 7.13 (d, *J* = 5.2 Hz, 1H, thiophene-H), 6.75 (d, *J* = 5.3 Hz, 1H, thiophene-H), 2.81 (t, *J* = 7.3 Hz, 2H, thiophene-C$\underline{H}_2$CH$_2$(CH$_2$)$_3$CH$_3$), 1.65 (q, *J* = 7.5 Hz, 2H, thiophene-CH$_2$C$\underline{H}_2$(CH$_2$)$_3$CH$_3$), 1.38 – 1.29 (m, 6H, thiophene-CH$_2$CH$_2$(C$\underline{H}_2$)$_3$CH$_3$), 0.93 – 0.86 (m, 3H, thiophene-CH$_2$CH$_2$(CH$_2$)$_3$C$\underline{H}_3$).

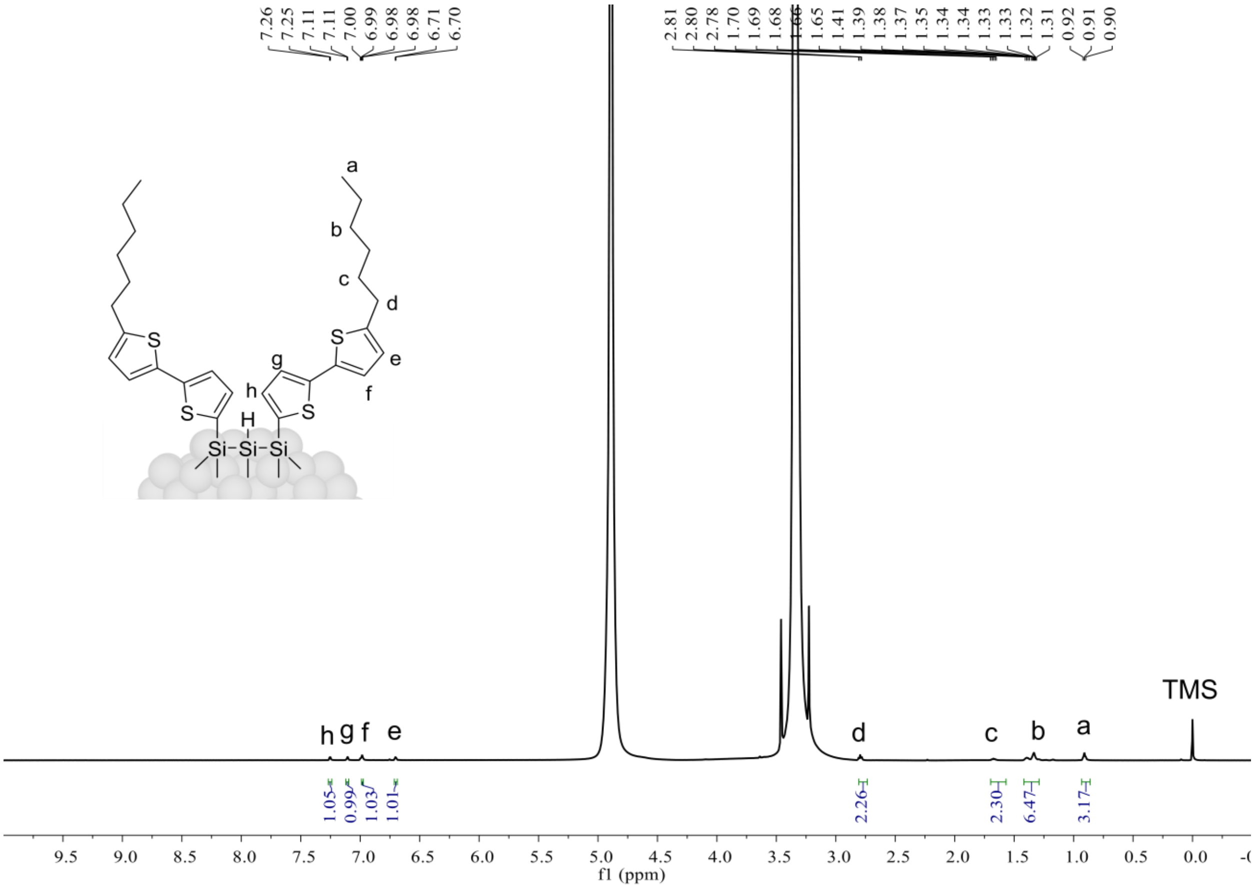

Supplementary Fig. 12 ^{1}H NMR spectrum of 2T-SiNC (600 MHz, methanol-*d*$_4$). δ 3.31 (residual chloroform), 4.87 (trace amount of H_2O in solvent), 7.25 (d, *J* = 5.1 Hz, 1H, thiophene-H), 7.11 (d, *J* = 3.6 Hz, 1H, thiophene-H), 6.98 (d, *J* = 3.6 Hz, 1H, thiophene-H), 6.70 (d, *J* = 3.5 Hz, 1H, thiophene-H), 2.79 (t, *J* = 7.6 Hz, 2H, thiophene-C$\underline{H}_2$$CH_2(CH_2)_3CH_3$), 1.67 (q, *J* = 7.6 Hz, 2H, thiophene-CH_2C$\underline{H}_2$$(CH_2)_3CH_3$), 1.42 – 1.32 (m, 6H, thiophene-CH_2CH_2(C$\underline{H}_2$)$_3CH_3$), 0.92 – 0.90 (q, *J* = 6.9 Hz, 3H, thiophene-$CH_2CH_2(CH_2)_3$C$\underline{H}_3$).

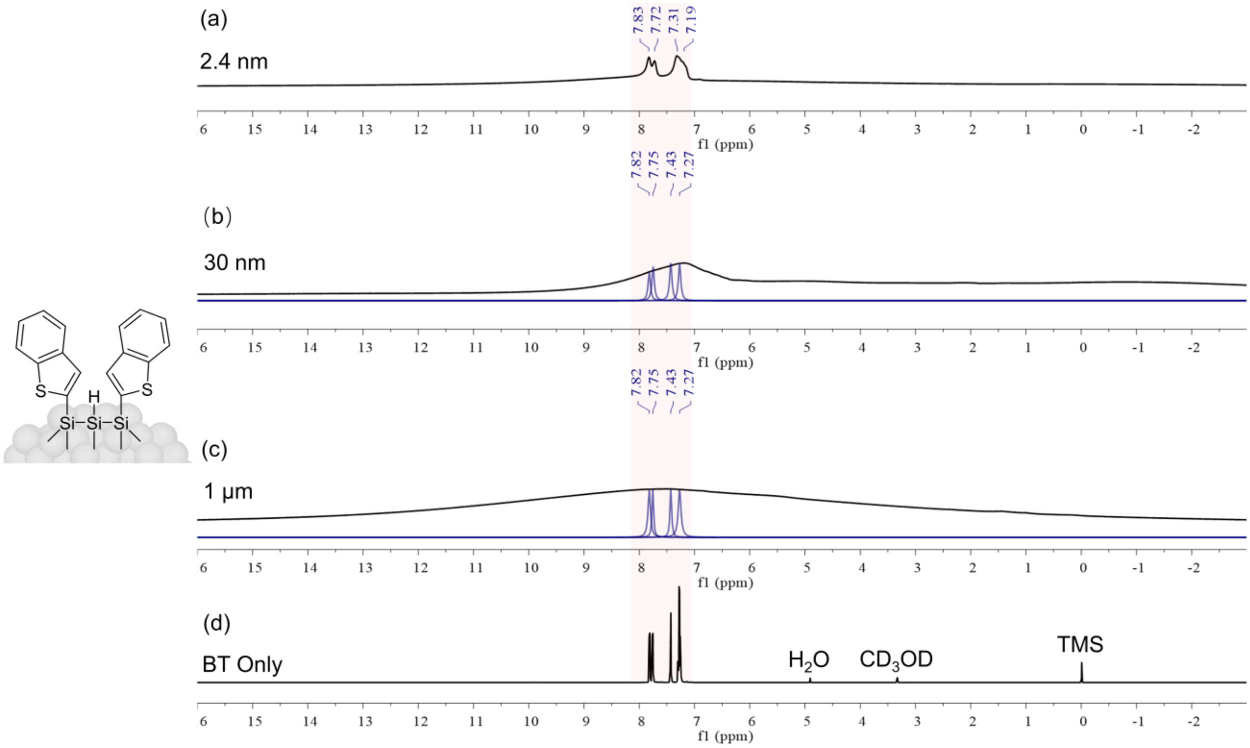

Supplementary Fig. 13 Solid-state ^{1}H NMR spectra of BT-SiNC with various sizes: (a) 2.4 nm, (b) 30 nm, (c) 1 μm, and (d) the freestanding BT molecule.

Supplementary Note 4 | Surface ligand coverage calculation.

The estimation of the ligand surface coverage on SiNCs was performed based on the TGA results following a published method.[11] The sample of 1T-passivated SiNC (d = 2.35 nm) was used as the calculation example (see below).

The detailed calculation employs the following parameters: N_A: Avogadro's number; $D_{\mathrm{Si\ atom}}$: the diameter of a silicon atom; V_{SiNC}: volume of a single SiNC; R_{SiNC}: radius of SiNC; m_{SiNC}: mass of a single SiNC; ρ_{Si}: density of diamond-structural silicon; n_{SiNC}: molar quantity of SiNC in the sample; M_{Si}: atomic mass of silicon; N_{SiNC}: total number of SiNC in the sample; R_{core}: the radius of the SiNC core (i.e., excluding the surface silicon atoms); V_{core}: core volume; m_{core}: core mass; n_{core}: mole of the core; N_{core}: total number of cores; M_{ligand}: molecular weight of the specific ligand; N_{surface}: number of silicon atoms on the sample SiNC surfaces; N_{ligand}: number of such ligand on the sample surfaces.

The volume of SiNC is calculated from the average radius of SiNCs obtained from the particle size distribution analysis of the TEM images shown in Supplementary Figure S14:

$$V_{SiNC} = \frac{4}{3}\,\pi\, {R_{SiNC}}^{3} = \frac{4}{3}\,\pi\,(1.17\ \mathrm{nm})^{3} = 6.71\times10^{-21}\ \mathrm{cm}^{3} \qquad (1)$$

The mass of a single SiNC is calculated based on the volume of SiNC and the density of diamond-structured silicon:

$$m_{SiNC} = \rho_{SiNC}\, V_{SiNC} = 2.329\,\frac{\mathrm{g}}{\mathrm{cm}^{3}\times6.71\times10^{-21}\ \mathrm{cm}^{3}} = 1.56\times10^{-20}\,\mathrm{g} \qquad (2)$$

The molar value of SiNC is therefore to be:

$$n_{SiNCs} = \frac{m_{SiNC}}{M_{Si}} = \frac{1.56\times10^{-20}\ \text{g}}{28.09\,\frac{\text{g}}{\text{mol}}} = 5.56\times10^{-22}\ \text{mol} \tag{3}$$

Based on n_{SiNCs}, we can estimate the average number of Si atoms on a single SiNC:

$$N_{SiNC} = n_{SiNC}\,N_A = 5.56\times10^{-22}\ \text{mol}\times6.022\times10^{23}\ \text{mol}^{-1} = 334.82 \tag{4}$$

Define the core of SiNC to be the volume of SiNCs excluding all Si atoms exposed on the top surfaces, then the radius of the SiNC core should be:

$$R_{core} = R_{SiNC} - D_{Si\ atom} = 1.17\ \text{nm} - 0.23\ \text{nm} = 0.94\ \text{nm} \tag{5}$$

and the volume of the SiNC core is:

$$V_{core} = \frac{4}{3}\,\pi\,{R_{core}}^{3} = \frac{4}{3}\,\pi\,(0.94\ \text{nm})^3 = 3.47\times10^{-21}\ \text{cm}^3 \tag{6}$$

Based on the core volume, we can calculate the average mass of the core:

$$m_{core} = \rho_{core}\,V_{core} = 2.329\,\frac{\text{g}}{\text{cm}^3\times3.47\times10^{-21}\ \text{cm}^3} = 8.08\times10^{-21}\ \text{g} \tag{7}$$

Then the molar value of the SiNC core is:

$$n_{core} = \frac{m_{core}}{M_{Si}} = \frac{8.08\times10^{-21}\ \text{g}}{28.09\,\frac{\text{g}}{\text{mol}}} = 2.88\times10^{-22}\ \text{mol} \tag{8}$$

and the corresponding average number of Si atoms used to construct the SiNC core is:

$$N_{core} = n_{core}\,N_A = 2.88\times10^{-22}\ \text{mol}\times6.022\times10^{23}\ \text{mol}^{-1} = 173.38 \tag{9}$$

Based on N_{SiNCs} and N_{core}, we can estimate the number of surface ligands on a single SiNC, which is equal to the number of surface Si atoms on a single SiNC (see Additional Note below for more information):

$$N_{Ligand} = N_{surface} = N_{SiNCs} - N_{core} = 334.82 - 173.38 = 161.44 \tag{10}$$

The weight loss of ideal model was calculated from the theoretical ligand mass divided by the sum of the theoretical ligand mass and the silicon core mass:

$$\%weight\ loss\ of\ ideal\ model = \frac{theoretical\ weight\ of\ ligands}{theoretical\ weight\ of\ ligands\ and\ SiNCs} \times 100 \quad (11)$$

$$= \frac{\frac{N_{ligand}\ M_{ligand}}{N_A}}{\frac{N_{ligand}\ M_{ligand}}{N_A} + \frac{N_{SiNC}\ M_{SiNC}}{N_A}} \times 100\% \quad (12)$$

$$= \frac{\frac{161.44 \times 168.30\ \mathrm{g/mol}}{6.022 \times 10^{23}\ \mathrm{mol^{-1}}}}{\frac{161.44 \times 168.30\ \mathrm{g/mol}}{6.022 \times 10^{23}\ \mathrm{mol^{-1}}} + \frac{334.82 \times 28.09\ \mathrm{g/mol}}{6.022 \times 10^{23}\ \mathrm{mol^{-1}}}} \times 100\% = 74.29\%$$

The actual surface ligand coverage can be calculated based on the comparison between the experimental weight loss obtained from TGA test (see Additional Note 2 below for more information) and the weight loss of ideal model:

$$\%Surface\ Coverage = \frac{\%experimental\ weight\ loss}{\%weight\ loss\ of\ ideal\ model} = \frac{100\% - 85.99\%}{74.29\%} \times 100\% \quad (13)$$

$$= \mathbf{18.86}\%$$

Additional Note:

1. All SiNCs are considered to be spherical, and all surface Si atoms are considered to be identical.

2. The ideal mode is defined as all Si atoms on the surface are anchored by 1T, reaching 100% coverage. The %experimental weight loss was obtained from TGA results (Figure 3e).

3. The summary of the key parameters, such as R_{SiNC}, N_{ligand}, M_{ligand}, and the results of calculated surface coverage for each SiNC sample, are available in Supplementary Table 3.

Supplementary Table 3 | Summary of the key parameters for estimation of the surface coverage of ligand passivated SiNCs.

Sample	R_{SiNC} (nm)	N_{ligand} (a. u.)	M_{ligand} (g mol^{-1})	Weight loss at 900 ºC shown in Figure 3e (%)	Estimated surface coverage (%)
1T-SiNC	1.17	161.44	168.30	14.01	18.86
BT-SiNC	1.19	166.21	134.20	10.96	15.81
2BT-SiNC	1.22	177.04	248.38	9.14	11.36

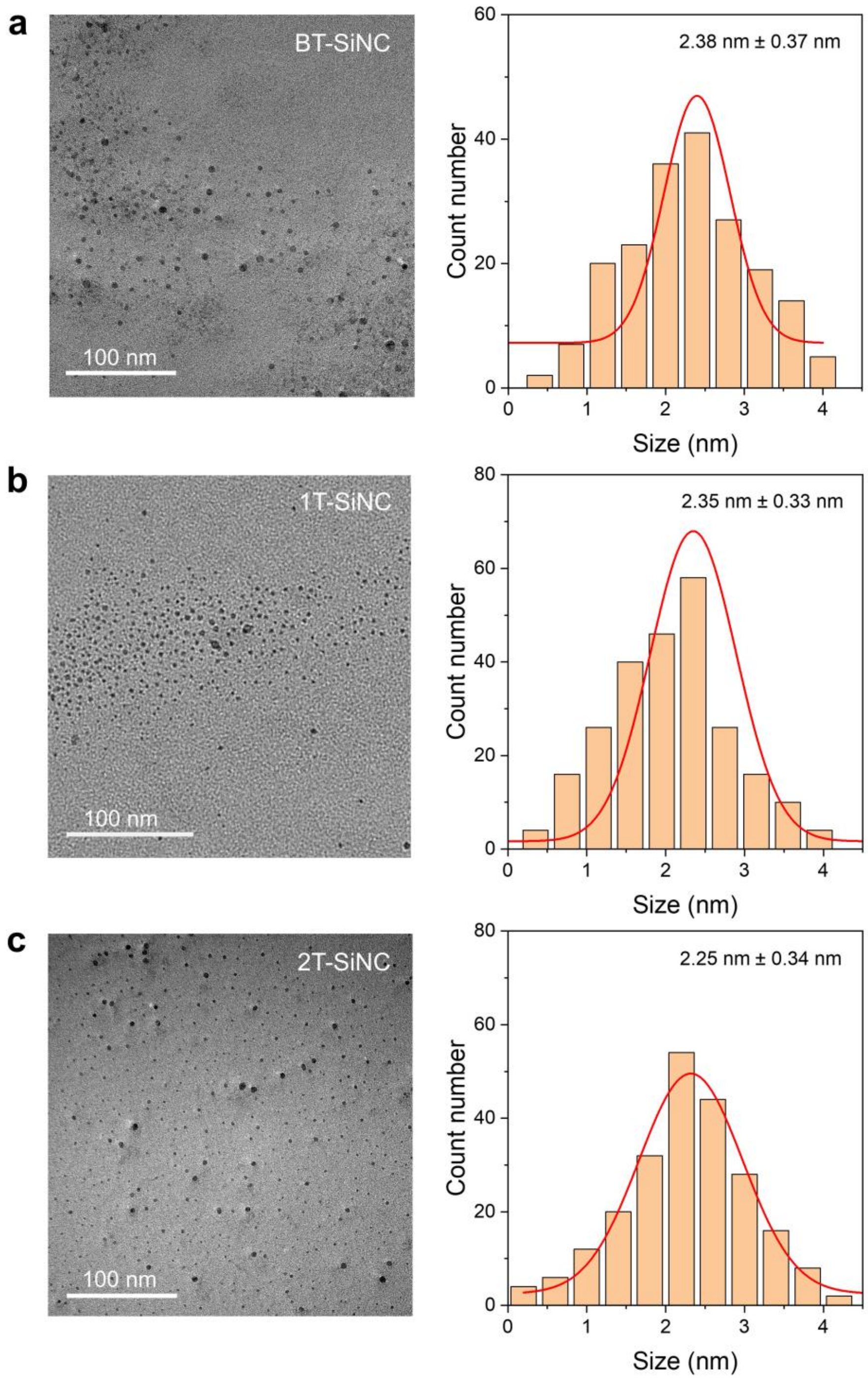

Supplementary Fig. 14 Bright-field TEM images and the corresponding particle size distribution analysis of (a) BT-, (b) 1T-, and (c) 2T-SiNCs.

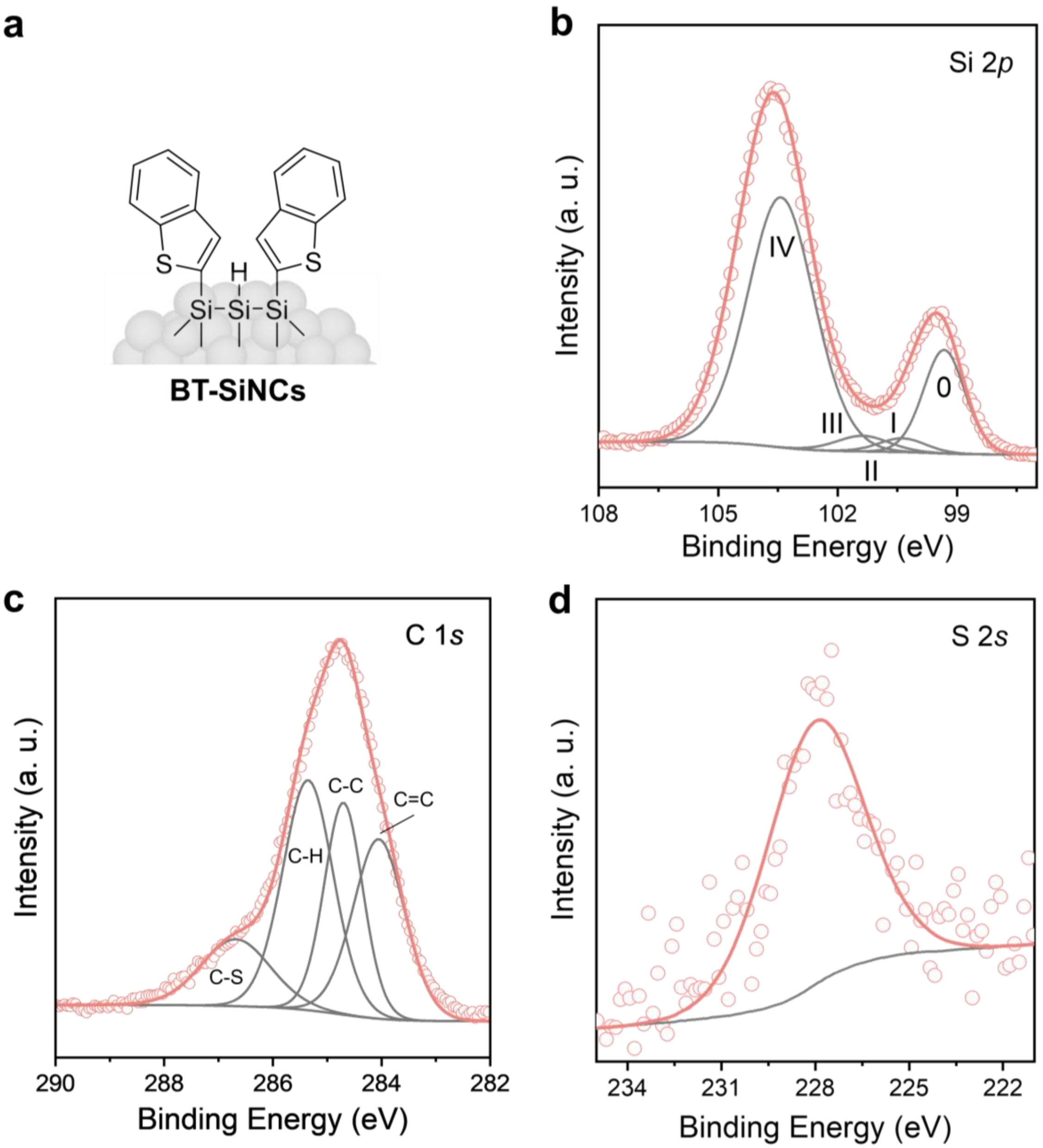

Supplementary Fig. 15 High-resolution X-ray photoelectron spectroscopic (XPS) results of BT-SiNC. (**a**) Model of BT-SiNC and the regions of (**b**) silicon 2*p*, (**c**) carbon 1*s*, and (**d**) sulfur 2*s*. Only the fitted Si $2p_{3/2}$ signals are present in Figure (**b**), and the Si $2p_{1/2}$ components are omitted for arity. C_{aryl} and C_{hetero} in Figure (**c**) represent the signals attributed to the carbon atoms on the aryl and thiophene groups, respectively.

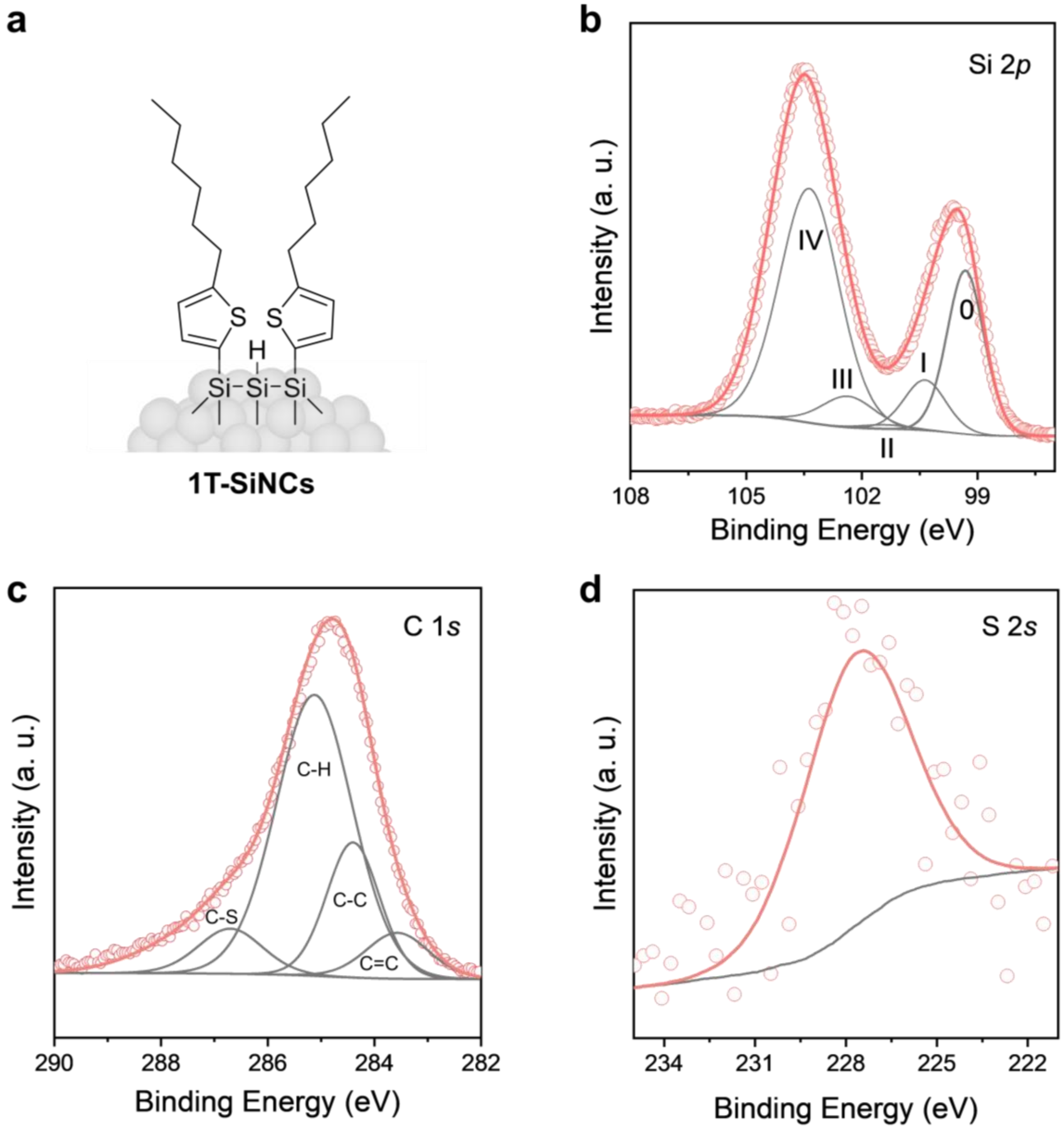

Supplementary Fig. 16 High-resolution X-ray photoelectron spectroscopic (XPS) results of 1T-SiNC. (**a**) Model of 1T-SiNC and the regions of (**b**) silicon 2*p*, (**c**) carbon 1*s*, and (**d**) sulfur 2*s*. Only the fitted Si $2p_{3/2}$ signals are present in Figure (**b**), and the Si $2p_{1/2}$ components are omitted for clarity. C=C, C-C, C-H, and C-S in Figure (**c**) represent the signals attributed to the carbon atoms on the *n*-hexyl and thiophene groups, respectively.

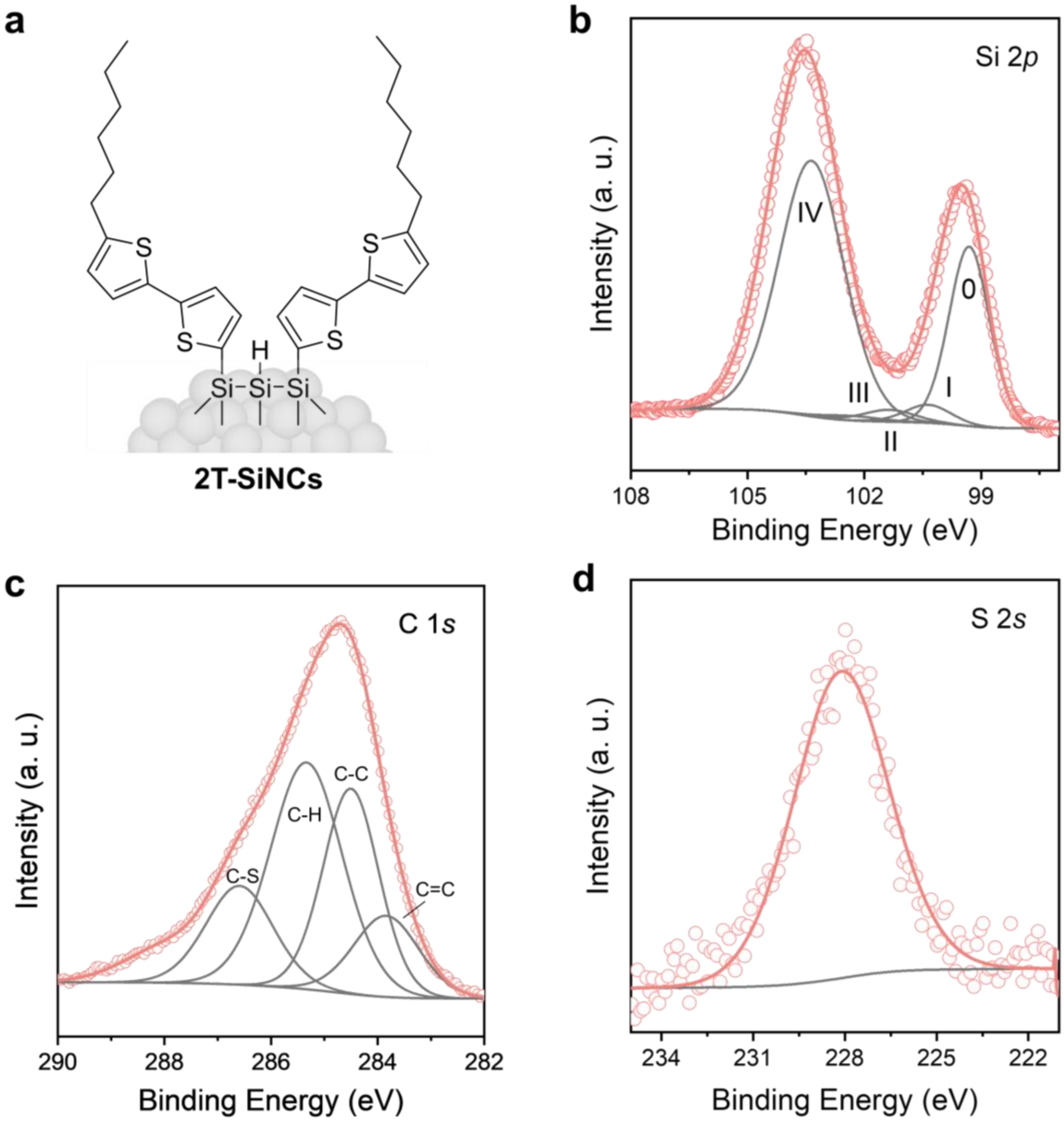

Supplementary Fig. 17 High-resolution X-ray photoelectron spectroscopic (XPS) results of 2T-SiNC. (**a**) Model of 2T-SiNC and the regions of (**b**) silicon 2*p*, (**c**) carbon 1*s*, and (**d**) sulfur 2*s*. Only the fitted Si $2p_{3/2}$ signals are present in Figure (**b**), and the Si $2p_{1/2}$ components are omitted for clarity. C=C, C-C, C-H, and C-S in Figure (**c**) represent the signals attributed to the carbon atoms on the *n*-hexyl and thiophene groups, respectively.

Supplementary Table 4 | PL lifetime decay fitting results of C8-SiNC, BT-SiNC, 1T-SiNC, 2T-SiNC, 2T, BT and 1T samples shown in Figure 4. The decay features were calculated according to the following equation:

$$y(t) = y_0 + A_1 e^{-\frac{x-x_0}{\tau_1}} + A_2 e^{-\frac{x-x_0}{\tau_2}} + \cdots + A_n e^{-\frac{x-x_0}{\tau_n}} \quad (14)$$

Sample	τ_1	τ_2	τ_3	τ_{avg}*
**C8-SiNC**	330.8 μs (58.09%)	695.2 μs (7.78%)	792.8 μs (33.76%)	514.1 μs
**BT-SiNC**	51.7 μs (32.87%)	281.4 μs (67.13%)	N/A**	205.9 μs
**1T-SiNC**	47.1 μs (36.35%)	131.2 μs (40.96%)	376.1 μs (22.69%)	156.2 μs
**2T-SiNC**	0.6 ns (94.92%)	3.2 ns (5.08%)	N/A**	0.8 ns
**2T only**	0.6 ns (98.79%)	7.2 ns (1.21%)	N/A**	0.7 ns
**BT only**	0.6 ns (85.04%)	14.9 ns (1.17%)	N/A**	1.1 ns
**1T only**	0.8 ns (39.52%)	4.9 ns (60.48%)	N/A**	3.3 ns

$$* \tau_{avg} = \frac{A_1\tau_1^2 + A_2\tau_2^2 + \cdots + A_n\tau_n^2}{A_1\tau_1 + A_2\tau_2 + \cdots + A_n\tau_n} \quad (15)$$

** The decay profiles exhibit double exponential features.

Supplementary Table 5 | Summary of PLQY values of C8-SiNC, BT-SiNC, 1T-SiNC, 2T-SiNC, 2T, BT and 1T.

**Sample**	Solvent	λ_{ex}	λ_{em} max	PLQY (%)
**C8-SiNC**	toluene	365 nm	939 nm	21.8
**BT-SiNC**	toluene	365 nm	936 nm	24.2
**1T-SiNC**	toluene	365 nm	825 nm	22.3
**2T-SiNC**	toluene	365 nm	408 nm	6.4
**2T only**	toluene	365 nm	415 nm	3.2
**BT only**	toluene	365 nm	406 nm	2.2
**1T only**	toluene	365 nm	422 nm	1.8

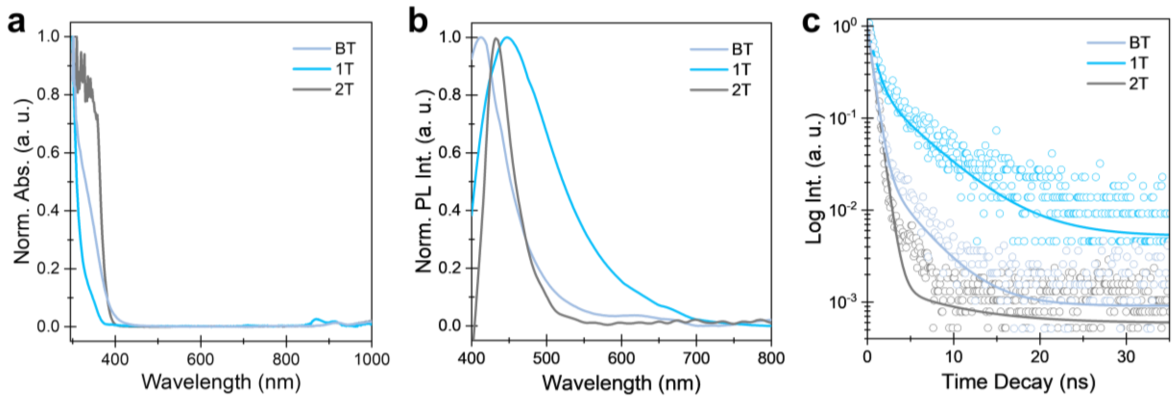

Supplementary Fig. 18 (a) Absorption and (b) PL spectra and (c) and the corresponding PL lifetime results of freestanding ligand molecules BT, 1T, and 2T. All samples were dispersed in toluene for the measurements in the air at room temperature.

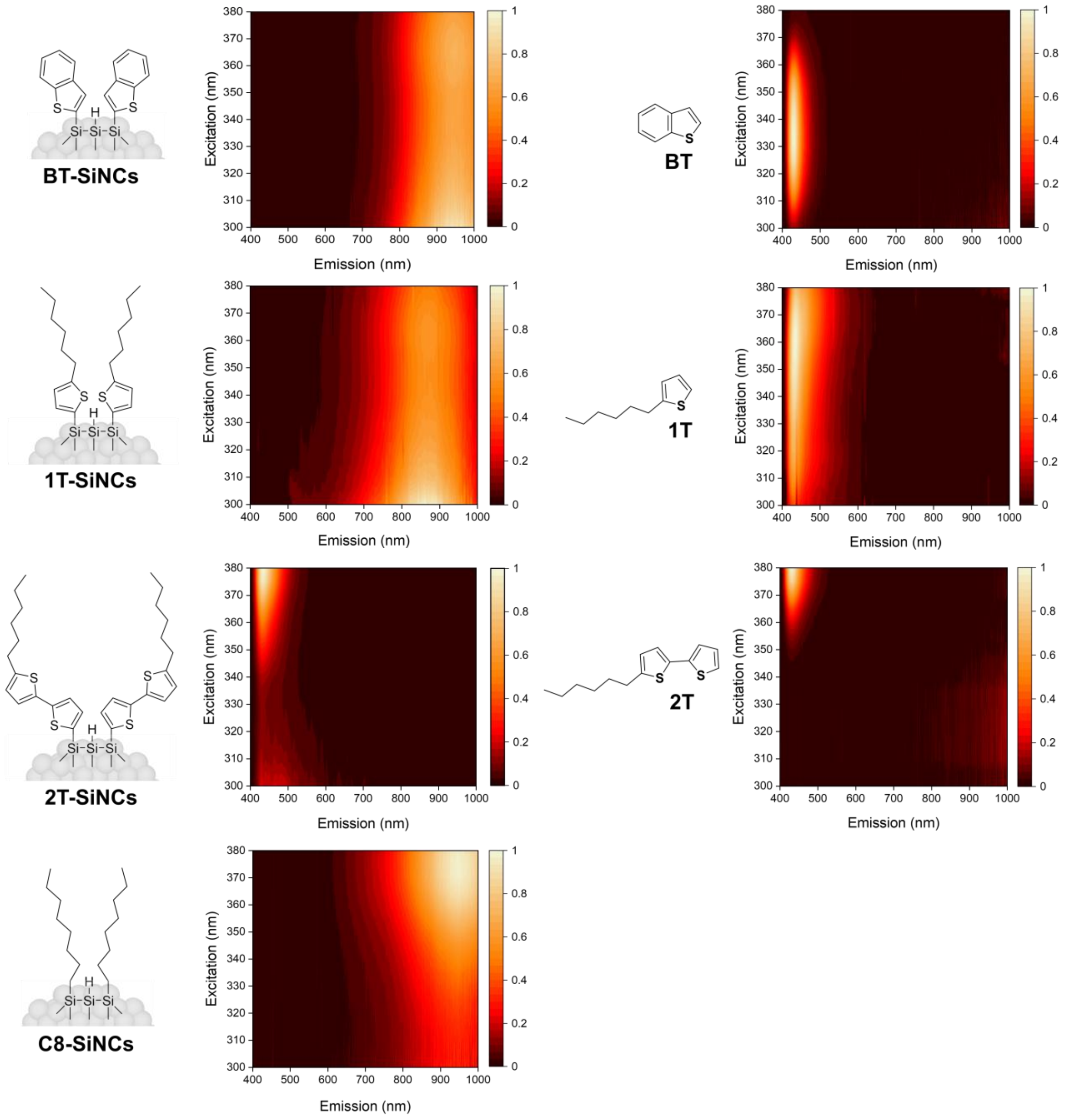

Supplementary Fig. 19 EEM results of BT-, 1T-, 2T-, and C8-SiNCs and freestanding ligand molecules (BT, 1T, and 2T). All samples were dispersed in toluene for the measurements in the air at room temperature.

Supplementary Note 5 | TD-DFT simulations and experimental results of the absorption features of freestanding thiophenes:

Geometry optimizations were performed using the Gaussian16 optimizer.[12] All geometry optimizations were computed using the functional M06-2X functional and the detailed configurations of the relaxed molecular structures are available in Supplementary Tables 6 - 8. The Def2-TZVP basis set was used for all the atoms. Frequency calculations at the same level of theory were performed to identify the number of imaginary frequencies (zero for local minimum and one for transition states). TD-DFT calculations were performed at the PBE0-SMD(toluene)/def2tzvp level of theory, and the simulated values are consistent with the experimental results as shown in Supplementary Figures 20 – 23.

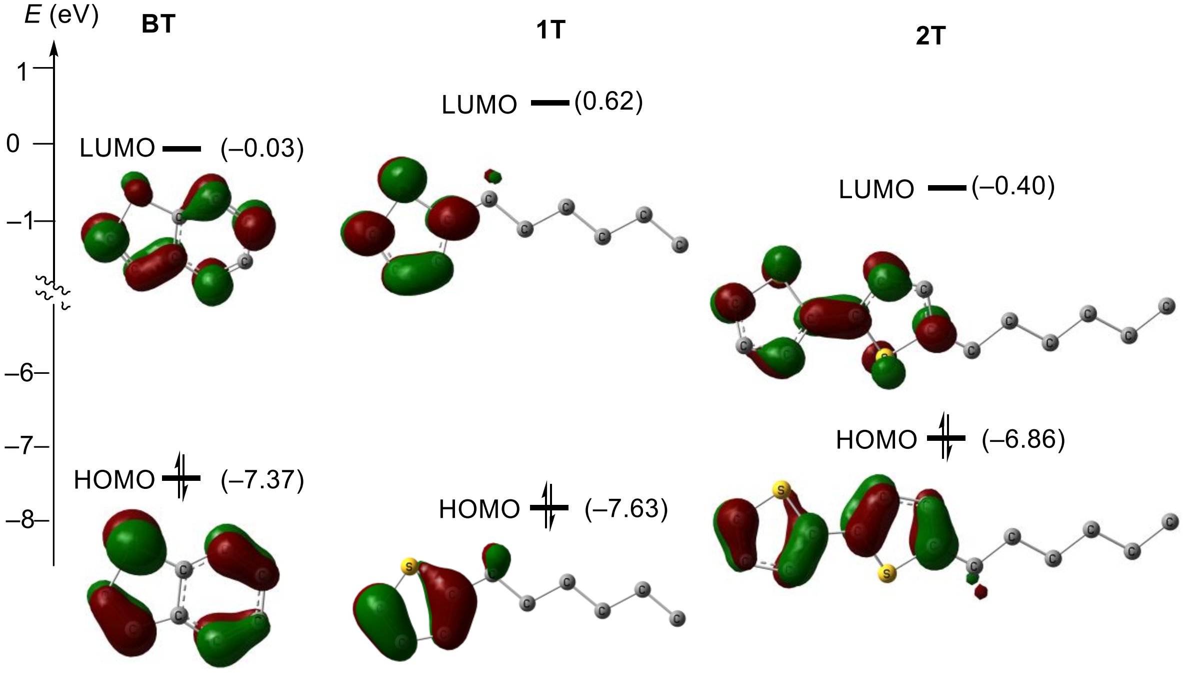

Supplementary Fig. 20 Energy diagram for the frontier Kohn-Sham orbitals (isovalue = 0.05) of compounds BT, 1T, and 2T.

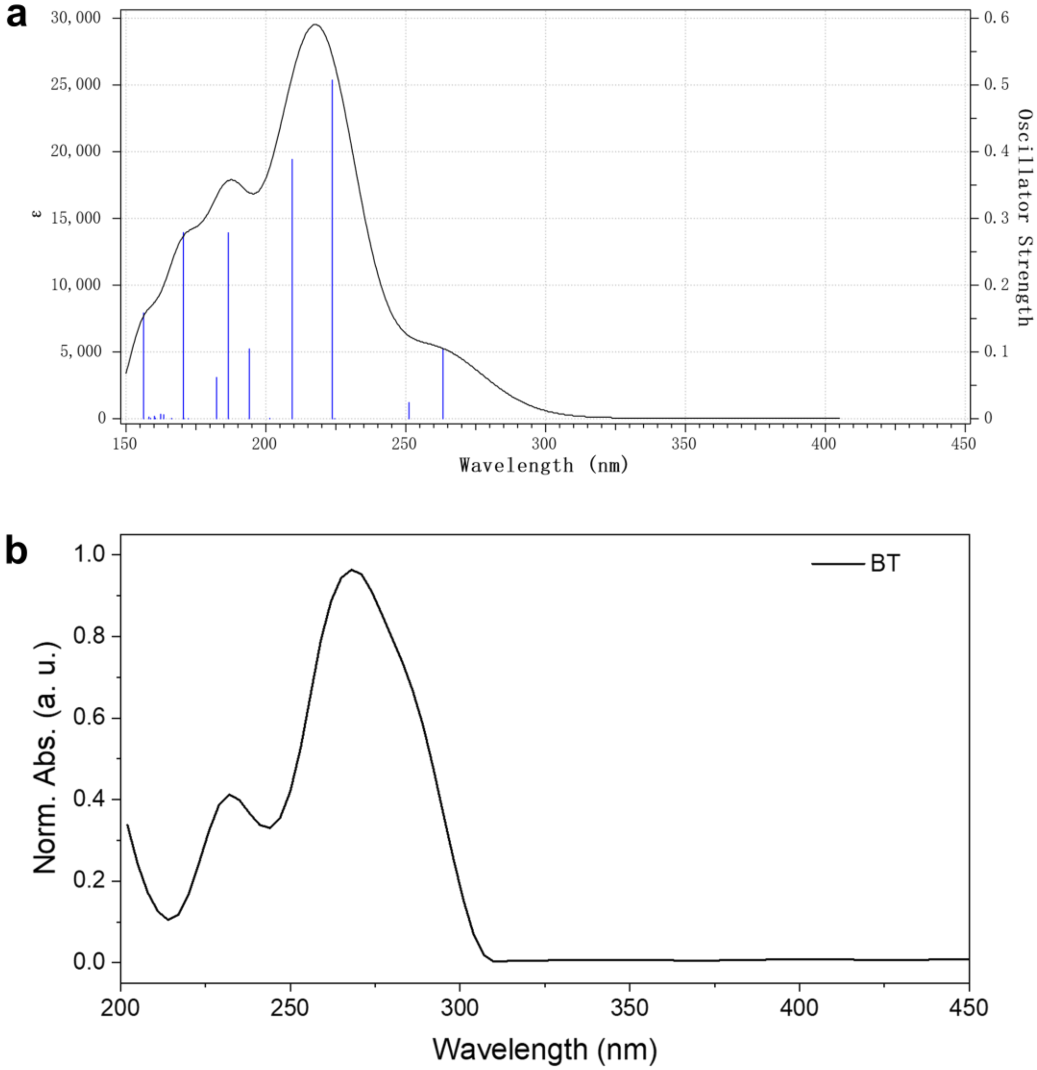

Supplementary Fig. 21 (**a**) Simulated UV-vis spectrum of the freestanding BT molecule from TD-DFT calculations (black line). Individual Gaussian absorption bands are shown in blue bars. The absorption at 263 nm is mainly attributed to the $\pi \rightarrow \pi^*$ transition (HOMO→LUMO, 76%). (b) Absorption spectrum of freestanding BT molecule dissolved in toluene.

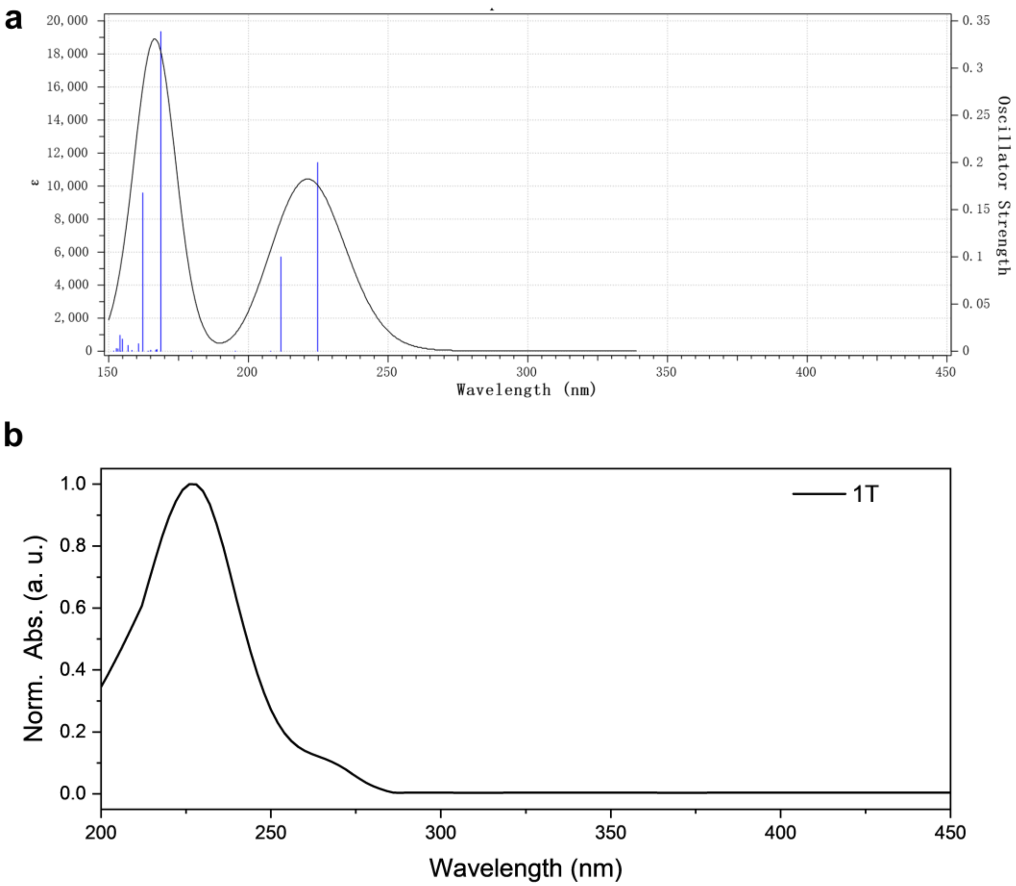

Supplementary Fig. 22 (**a**) Simulated UV-vis spectrum of the freestanding 1T molecule from TD-DFT calculations (black line). Individual Gaussian absorption bands are shown in blue bars. The absorption at 225 nm is attributed to the π→π* transition (HOMO→LUMO, 97%). (**b**) Absorption spectrum of freestanding 1T molecule dissolved in toluene.

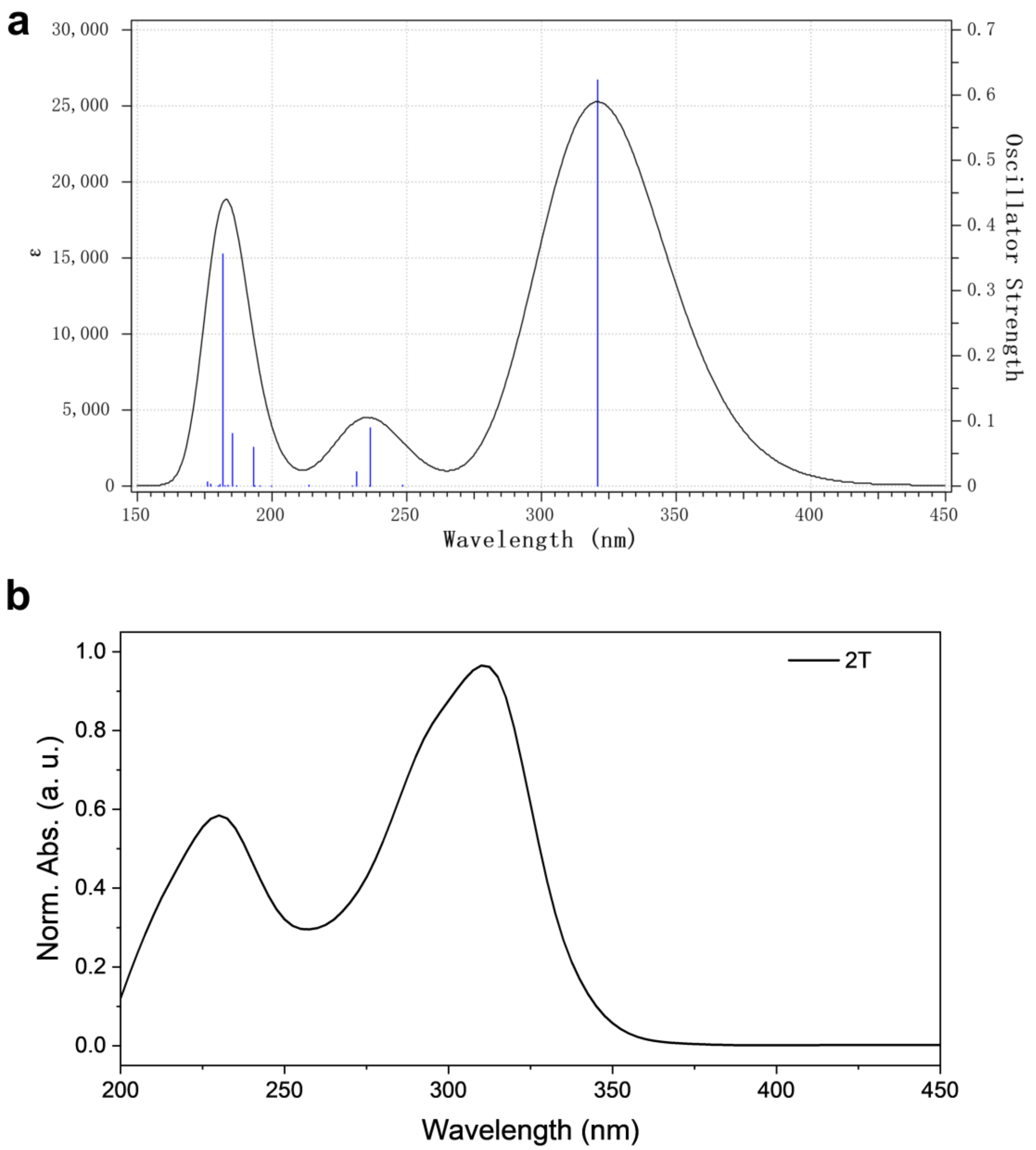

Supplementary Fig. 23 (**a**) Simulated UV-vis spectrum of the freestanding 2T molecule from TD-DFT calculations (black line). Individual Gaussian absorption bands are shown in blue bars. The absorption at 321 nm is attributed to the π→π* transition (HOMO→LUMO, 99%). (**b**) Absorption spectrum of freestanding 2T molecule dissolved in toluene.

Supplementary Table 6 | BT molecule as model parameters used for TD-DFT calculations.

Element	Coordination (x, y, z)		
C	0.00000000	-0.55328348	0.0000000
C	0.00432105	0.85325249	-0.0000000
C	1.23223960	1.52779449	-0.0000000
C	2.40463627	0.80461121	-0.0000000
C	2.38195815	-0.59754201	-0.0000000
C	1.18691622	-1.28590094	-0.0000000
C	-2.27226626	0.42360507	0.0000000
C	-1.32866037	1.38846883	0.0000000
H	1.25128428	2.61049723	-0.0000000
H	3.35545139	1.32093616	-0.0000000
H	3.31393325	-1.14721302	-0.0000000
H	1.16945532	-2.36789924	-0.0000000
H	-1.55037747	2.44601417	0.0000000
S	-1.61573671	-1.17387977	0.0000000
H	-3.34282719	0.55370708	0.0000000

Supplementary Table 7 | 1T molecule as model parameters used for TD-DFT calculations.

Element	Coordination (x, y, z)		
C	-4.25012526	0.40050108	-0.00020687
C	-3.47936387	1.51851535	0.00005108
C	-2.08643244	1.21996878	0.00016424
C	-1.82215083	-0.11691038	0.00006806
S	-3.28999748	-1.01668897	-0.00008314
H	-5.32540975	0.33041970	-0.00038353
H	-3.88287765	2.52060164	0.00009441
H	-1.31238818	1.97393049	0.00030423
C	-0.49531578	-0.81811021	0.00022127
H	-0.43161649	-1.47344266	0.87437510
H	-0.43164002	-1.47389532	-0.87359241
C	0.69575037	0.13222545	-0.00002645
H	0.64151654	0.78399523	0.87711165
H	0.64143172	0.78364494	-0.87741895
C	2.02722303	-0.60800975	0.00007566
H	2.07883879	-1.26335193	-0.87584856
H	2.07885267	-1.26308404	0.87619976
C	3.22882835	0.32805111	-0.00007336
H	3.17792345	0.98406923	0.87564079
H	3.17789848	0.98382536	-0.87596855
C	4.56335182	-0.40742484	0.00001196
H	4.61229538	-1.06206936	-0.87505097
H	4.61230339	-1.06185143	0.87523759
C	5.75545151	0.54056688	-0.00010989
H	5.73933531	1.18524340	0.88076670
H	6.70087070	-0.00228501	-0.00005075
H	5.73932388	1.18503241	-0.88114054

Supplementary Table 8 | 2T molecule as model parameters used for TD-DFT calculations.

Element	Coordination (x, y, z)		
C	5.95549600	0.34621800	0.0624100
C	5.64718700	-0.95400700	-0.1926710
C	4.24766900	-1.17600300	-0.2658180
C	3.51421700	-0.03899400	-0.0637560
S	4.55037300	1.30604700	0.2285610
H	6.93085900	0.79276500	0.1641990
H	6.38951600	-1.72642900	-0.3296490
H	3.79533700	-2.13505200	-0.4767420
C	2.07108200	0.12140600	-0.0704490
C	1.33739500	1.26057000	-0.2322430
S	1.02938200	-1.23754500	0.1479800
C	-0.06448000	1.03224400	-0.1860540
H	1.78724400	2.23020200	-0.3963290
C	-0.39296700	-0.27553300	0.0073830
H	-0.80093100	1.81470400	-0.2994440
C	-1.74995000	-0.90684800	0.1045200
H	-1.84996300	-1.67201400	-0.6717170
H	-1.83704300	-1.43378700	1.0599340
C	-2.89538200	0.08995200	-0.0241040
H	-2.81961900	0.60974000	-0.9838830
H	-2.80223500	0.85370400	0.7537870
C	-4.25960200	-0.57957900	0.0833470
H	-4.33305500	-1.10279100	1.0426230
H	-4.34986400	-1.34746600	-0.6922820
C	-5.41657800	0.40303900	-0.0439930
H	-5.34415300	0.92657600	-1.0034180
H	-5.32656700	1.17195600	0.7309310
C	-6.78354400	-0.26158700	0.0639080
H	-6.85390800	-0.78380400	1.0225820

H	-6.87139200	-1.02938300	-0.7103760
C	-7.93064800	0.73212700	-0.0653260
H	-7.89385200	1.24490700	-1.0284110
H	-8.89996100	0.23956000	0.0145440
H	-7.87585500	1.49255100	0.7160680

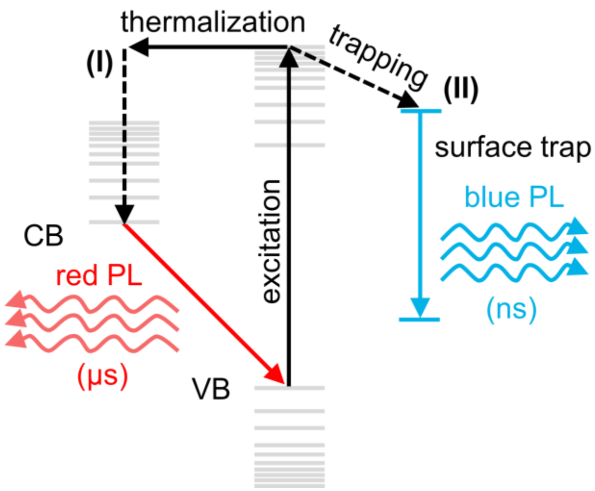

Supplementary Fig. 24 Proposed origin of the blue emission of 2T-SiNCs. Surface states are introduced by surface dehydrocoupling in which the photoexcited charge carriers recombine through the radiative recombination at the band edge (Channel I, e.g., the cases of BT- and 1T-SiNCs) and radioactive surface state (Channel II, e.g., the case of 2T-SiNC).

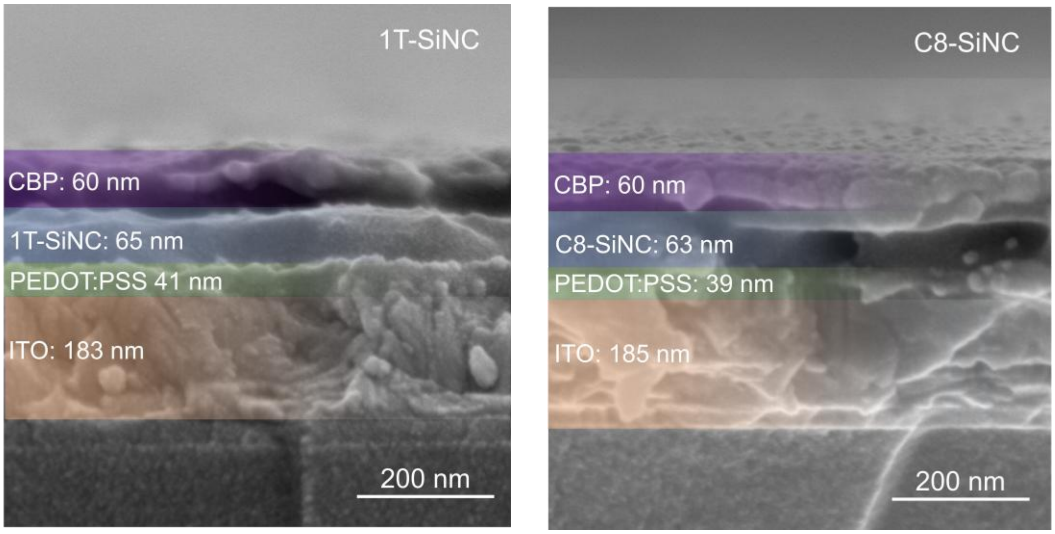

Supplementary Fig. 25 Cross-sectional SEM images the hole-only devices using 1T-SiNC and C8-SiNC as the active materials.

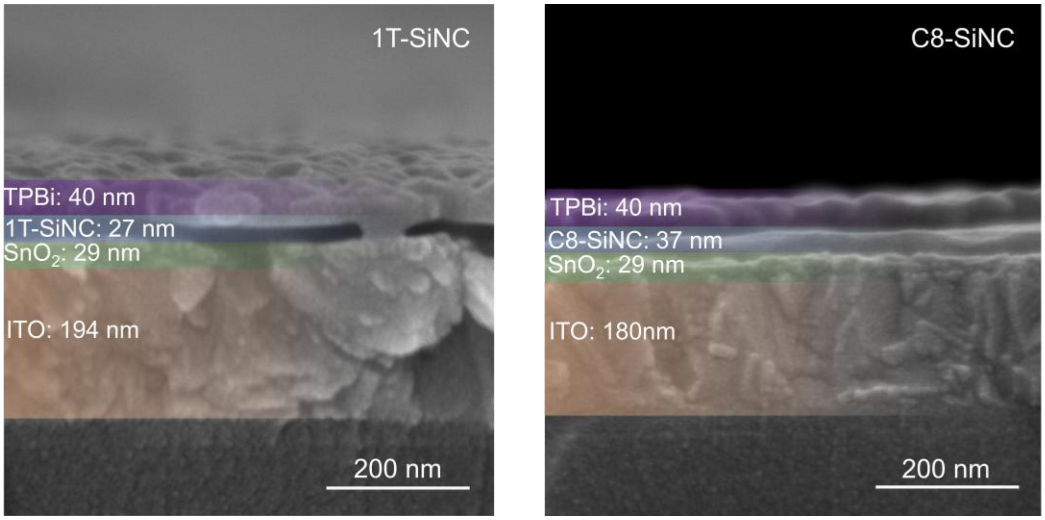

Supplementary Fig. 26 Cross-sectional SEM images the electron-only devices using 1T-SiNC and C8-SiNC as the active materials.

Supplementary Table 9 | Summary of the carrier mobilities and trap densities of 1T-SiNC and C8-SiNC obtained from SCLC measurements.

	μ_e (cm^2 V^{-1} s^{-1})	μ_h (cm^2 V^{-1} s^{-1})	n_{te} (cm^{-3})	n_{th} (cm^{-3})
1T-SiNC	6.47×10^{-6}	1.52×10^{-5}	8.78×10^{15}	3.07×10^{17}
C8-SiNC	3.16×10^{-8}	9.04×10^{-8}	1.70×10^{16}	6.29×10^{17}

Supplementary Table 10 | Summary of SCLC data for SiNC surfaces functionalized with various ligands in literature.

ligand	Particle size (nm)	μ_e (cm^2 V^{-1} s^{-1})	μ_h (cm^2 V^{-1} s^{-1})	Ref.# in ESI
P3HT	3	/	1.1×10^{-9}	13
TTQ	7	1.0×10^{-6}	/	14
C8	3	6.57×10^{-7}	6.56×10^{-9}	15
PhPr	3	1.31×10^{-8}	1.57×10^{-8}	15

Note: P3HT = Poly(3-hexylthiophene-2,5-diyl); C8 = Octyl; TTQ = 2,3,5,6-Tetrafluoro-7,7,8,8-tetracyano-quinodimethane; PhPr = Phenylpropyl.

Supplementary References

1. Gu, J. & Cai, C. Stereoselective synthesis of vinylsilanes via copper-catalyzed silylation of alkenes with silanes. *Chem. Commun.* **52**, 10779-10782, (2016).
2. Guo, L. et al. Decarbonylative silylation of esters by combined nickel and copper catalysis for the synthesis of arylsilanes and heteroarylsilanes. *Angew. Chem. Int. Ed.* **55**, 11810-11813, (2016).
3. Lu, B. & Falck, J. R. Efficient iridium-catalyzed C-H functionalization/silylation of heteroarenes. *Angew. Chem. Int. Ed.* **47**, 7508-7510, (2008).
4. Miura, H. et al. Electrophilic C(sp2)-H silylation by supported gold catalysts. *ChemCatChem* **13**, 4705-4713, (2021).
5. Nozawa-Kumada, K. et al. Deprotonative silylation of aromatic C-H bonds mediated by a combination of trifluoromethyltrialkylsilane and fluoride. *J. Org. Chem.* **82**, 9487-9496, (2017).
6. Rubio-Pérez, L. et al. A well-defined NHC-Ir(III) catalyst for the silylation of aromatic C-H bonds: Substrate survey and mechanistic insights. *Chem. Sci.* **8**, 4811-4822, (2017).
7. Wang, X. et al. Nickel/copper-cocatalyzed decarbonylative silylation of acyl fluorides. *Chem. Commun.* **55**, 10507-10510, (2019).
8. Xu, W. et al. Rare-earth-catalyzed C-H silylation of aromatic heterocycles with hydrosilanes. *Chem. Asian J.* **15**, 753-756, (2020).
9. Swann, N. et al. Intermolecular C-H silylations of arenes and heteroarenes with mono-, bis-, and tris(trimethylsiloxy)hydrosilanes: Control of silane redistribution under operationally diverse approaches. *Chem. Sci.* **15**, 11912-11918, (2024).
10. Toutov, A. A. et al. Silylation of C-H bonds in aromatic heterocycles by an earth-abundant metal catalyst. *Nature* **518**, 80-84, (2015).
11. Lai, M. et al. Direct arylation of silicon nanocrystals with hexadehydro-diels-alder-derived benzynes. *Angew. Chem. Int. Ed.* **62**, e202304056 (2023).

12. M. J. Frisch, G. W. Trucks, H. B. Schlegel, G. E. Scuseria, M. A. Robb, J. R. Cheeseman, G. Scalmani, V. Barone, G. A. Petersson, H. Nakatsuji, X. Li, M. Caricato, A. V. Marenich, J. Bloino, B. G. Janesko, R. Gomperts, B. Mennucci, H. P. Hratchian, J. V. Ortiz, A. F. Izmaylov, J. L. Sonnenberg, D. Williams-Young, F. Ding, F. Lipparini, F. Egidi, J. Goings, B. Peng, A. Petrone, T. Henderson, D. Ranasinghe, V. G. Zakrzewski, J. Gao, N. Rega, G. Zheng, W. Liang, M. Hada, M. Ehara, K. Toyota, R. Fukuda, J. Hasegawa, M. Ishida, T. Nakajima, Y. Honda, O. Kitao, H. Nakai, T. Vreven, K. Throssell, J. A. Montgomery Jr., J. E. Peralta, F. Ogliaro, M. J. Bearpark, J. J. Heyd, E. N. Brothers, K. N. Kudin, V. N. Staroverov, T. A. Keith, R. Kobayashi, J. Normand, K. Raghavachari, A. P. Rendell, J. C. Burant, S. S. Iyengar, J. Tomasi, M. Cossi, J. M. Millam, M. Klene, C. Adamo, R. Cammi, J. W. Ochterski, R. L. Martin, K. Morokuma, O. Farkas, J. B. Foresman, D. J. Fox, Gaussian 16, Revision C.01 ed., Gaussian, Inc., Wallingford CT, 2016.

13. Liu, C. Y. Holman, Z. C. & Kortshagen, U. R. Optimization of SiNC/P3HT hybrid solar cells. *Adv. Funct. Mater.* **20**, 2157–2164 (2010).

14. Pereira, R. N. et al. Resonant electronic coupling enabled by small molecules in nanocrystal solids. *Nano Lett.* **14**, 3817–3826 (2014).

15. Liu, X. et al. Light-Emitting Diodes based on colloidal silicon quantum dots with octyl and phenylpropyl ligands. *ACS Appl. Mater. Interfaces* **10**, 5959-5966, (2018).
